# PLEKHA4 is a novel prognostic biomarker that reshapes the tumor microenvironment in lower-grade glioma

**DOI:** 10.3389/fimmu.2023.1128244

**Published:** 2023-09-25

**Authors:** Wenqian Zhi, Ye Wang, Chenyu Jiang, Yuqin Gong, Qiuyan Chen, Xiang Mao, Wensheng Deng, Shasha Zhao

**Affiliations:** ^1^College of Life Science and Health, Wuhan University of Science and Technology, Wuhan, Hubei, China; ^2^Institute of Hygiene Toxicology, Wuhan Centre for Disease Prevention and Control, Wuhan, Hubei, China

**Keywords:** lower-grade glioma, PLEKHA4, tumor microenvironment, prognosis, therapy

## Abstract

**Background:**

Lower-grade glioma (LGG) is a primary intracranial tumor that carry a high risk of malignant transformation and limited therapeutic options. Emerging evidence indicates that the tumor microenvironment (TME) is a superior predictor for tumor progression and therapy response. PLEKHA4 has been demonstrated to be a biomarker for LGG that correlate with immune infiltration. However, the fundamental mechanism by which PLEKHA4 contributes to LGG is still poorly understood.

**Methods:**

Multiple bioinformatic tools, including Tumor Immune Estimation Resource (TIMER), Gene Expression Profiling Interactive Analysis (GEPIA2), Shiny Methylation Analysis Resource Tool (SMART), etc., were incorporated to analyze the PLEKHA4. ESTIMATE, ssGSEA, CIBERSORT, TIDE and CellMiner algorithms were employed to determine the association of PLEKHA4 with TME, immunotherapy response and drug sensitivities. Immunohistochemistry (IHC)-based tissue microarrays and M2 macrophage infiltration assay were conducted to verify their associations.

**Results:**

PLEKHA4 expression was found to be dramatically upregulated and strongly associated with unfavorable overall survival (OS) and disease-specific survival (DSS) in LGG patients, as well as their poor clinicopathological characteristics. Cox regression analysis identified that PLEKHA4 was an independent prognostic factor. Methylation analysis revealed that DNA methylation correlates with PLEKHA4 expression and indicates a better outcome in LGG. Moreover, PLEKHA4 was remarkably correlated with immune responses and TME remodeling, as evidenced by its positive correlation with particular immune marker subsets and the putative infiltration of immune cells. Surprisingly, the proportion of M2 macrophages in TME was strikingly higher than others, inferring that PLEKHA4 may regulate the infiltration and polarization of M2 macrophages. Evidence provided by IHC-based tissue microarrays and M2 macrophage infiltration assay further validated our findings. Moreover, PLEKHA4 expression was found to be significantly correlated with chemokines, interleukins, and their receptors, further supporting the critical role of PLEKHA4 in reshaping the TME. Additionally, we found that PLEKHA4 expression was closely associated with drug sensitivities and immunotherapy responses, indicating that PLEKHA4 expression also had potential clinical significance in guiding immunotherapy and chemotherapy in LGG.

**Conclusion:**

PLEKHA4 plays a pivotal role in reshaping the TME of LGG patients, and may serve as a potential predictor for LGG prognosis and therapy.

## Introduction

Glioma, characterized by its high mortality and morbidity, is one of the most common primary intracranial malignancies in adults (1, 2). Classically, based on 2016 WHO histopathological grading system, gliomas of adult can be classified as grade II to IV (3). Glioblastoma multiforme (GBM), or WHO grade IV glioma, is deemed as the most aggressive and deadliest form, with a dismal 2-year survival rate of 26-33% (4). Gliomas of WHO grades II and III, including subtypes of astrocytomas, oligodendrogliomas, and oligoastrocytomas, are designated as low-grade gliomas (LGG) (5). Although LGG patients have a relatively better prognosis than those of GBMs, nearly 70% of LGG patients are prone to transform into high grade gliomas (defined as secondary GBM), which appear to be more aggressive with significantly poorer clinical outcomes (the median overall survival is about 7.8 months) than primary GBM (6–8).

Many molecular changes are recognized as predictors and prognostic indicators for LGG. It has been confirmed that biomarkers including S100A, TEAD4, SRSF9, METTL7B, CSPP1, and CKS2 have significant relevance to the diagnosis and prognosis of LGG patients (9–14). In addition, iron metabolism-related genes, cuproptosis-related genes, necroptosis-related gene, autophagy-related signatures, as well as autophagy related DNA methylation signature, are also important prognostic factors for LGG (15–19). However, these clinical pathologic and genetic factors used for predicting LGG in clinical practice is poor, which limits their early diagnosis and treatment. Hence, it is particular important to identify robust yet feasible cell-type-specific biomarkers to better guide the prognosis and therapy for LGG patients.

Current treatment for LGG favors maximum resection with consideration of combined chemoradiation for patients deemed “high risk”. Even though LGG patients are relatively sensitive to radiation and chemotherapy, the curative effect varies among individuals, and patient outcomes remain limited (7). Immunotherapy, mainly including immune checkpoint inhibitors (ICIs) that targeting cytotoxic T lymphocyte-associated antigen-4 (CTLA-4), programmed death-1 (PD-1), and programmed death ligand-1 (PD-L1), has become a promising and effective strategy with the ability to penetrate the blood-brain barrier (1, 20). A growing body of literature reported that immunotherapy efficacy and patient outcomes were closely related to the surrounding tumor microenvironment (TME), which is a complex and dynamic ecosystem consist of tumor cells, infiltrating immune cells, cancer-associated fibroblasts (CAFs), extracellular matrix (ECM), the tumor-related soluble factors, etc. (2, 21, 22). To be specific, tumor-associated macrophages (TAMs), including antitumor M1 macrophage or protumor M2 macrophage, are diverse and plastic under different stimulation in TME. Massive infiltration of M2 macrophages or enrichment of M2-related factors usually indicates cancer progression and a poor prognosis in LGG patients (23, 24). In addition, recent studies have revealed that, by remodeling ‘stroma’, CAFs play indispensable roles in conferring resistance to immune-based therapies (25–27). Moreover, TAMs and CAFs are also key players that give rise to cancer-induced immunosuppression, drug resistance, and tumor recurrence (23, 28, 29). Therefore, identification of specific yet robust biomarkers that may affect TME formation in LGG patients is of prominent significance, so as to predict patient prognosis as well as their potential response to specific therapies, allowing clinicians to identify individualized therapy for each patient.

In our original study, three GEO datasets (GSE44971, GSE109857, GSE116520) were analyzed to obtain differentially expressed genes (DEGs) between glioma and normal tissues. 52 overlapping DEGs from the three GEO datasets were screened. Protein-protein interaction enrichment analysis were performed using the online tool Metascape, and identified PLEKHA4 as a hub gene. PLEKHA4 (Pleckstrin Homology Domain Containing A4), also known as PEPP1 (Phosphoinositol 3-phosphate-binding protein 1), is a 779 amino acid protein that contains one pleckstrin homology (PH) domain, which is found in proteins that are involved in intracellular signaling. Previous studies have demonstrated that PLEKHA4 is a key modulator of Wnt and PCP signaling pathways through its function as an adaptor that tunes CUL3-KLHL12 activity at the plasma membrane (30). Moreover, PLEKHA4 was demonstrated to exert a potent effect on controlling melanoma proliferation through promoting Wnt/β-catenin signaling-mediated G1/S transition, and may act as a new avenue for the development of targeted therapies (31). Recently, PLEKHA4 has been reported to be upregulated and closely associated with immune infiltration in LGG patients (32, 33). However, the relationship among PLEKHA4 expression, DNA methylation signature, and clinical prognosis of LGG patients, as well as the biological role of PLEKHA4 in reshaping TME, are still not well understood. Whether PLEKHA4 could affect the immune response and drug sensitivities of LGG patients is still unclear.

To this end, we identified the expression profile and prognostic significance of PLEKHA4 in gliomas based on public data as well as clinical samples. Next, a prognostic model using identified independent prognostic factors was conducted. Calibration and Decision Curve Analysis (DCA) were subsequently performed to assess the clinical performance of the model. Then, genetic mutation, methylation alteration and coexpression network of PLEKHA4 in LGG were further explored. Gene Ontology (GO), Kyoto Encyclopedia of Genes and Genomes (KEGG) and Gene Set Enrichment Analysis (GSEA) were used to investigate the associated biological processes and pathways. Additionally, the potential link between PLEKHA4 expression and infiltrating immune cells, CAFs, as well as tumor-related soluble factors, was examined through the ESTIMATE, ssGSEA, TIMER and CIBERSORT algorithm. Experimental validations using THP-1-derived M2 macrophages and tissue microarrays confirmed the vital role of PLEKHA4 in regulating M2 macrophage polarization and infiltration. Finally, the clinical significance of PLEKHA4 in predicting the immunotherapy response and drug sensitivities was analyzed. Our research has discovered that PLEKHA4 may serve as an independent prognostic indicator, and shed light on the cellular and molecular basis of immune microenvironment in LGG patients, thereby providing an important basis for the evaluation of the clinical efficacy of immunotherapy and chemotherapy in LGG.

## Materials and methods

### Data acquisition

The mRNA-seq data used in pan-cancer analysis were downloaded from UCSC XENA database (https://xenabrowser.net), which provided unified RNA-seq data from the Cancer Genome Atlas (TCGA) and Genotype-Tissue Expression (GTEx) that were processed uniformly using Toil process, removing batch effects between databases (34). Principal Component Analysis (PCA) was performed to assess the contribution of any confounding factors and check whether the batch effects have been removed successfully. The mRNA-seq data and clinicopathological data of all glioma cases were retrieved from TCGA (http://cancergenome.nih.gov) and the Chinese Glioma Genome Atlas (CGGA) (http://www.cgga.org.cn/). In total, 528 LGG patients and 168 GBM patients from TCGA database were defined as training cohorts. 443 LGG patients and 249 GBM patients from CGGA database (mRNAseq_693), as well as 182 LGG patients and 139 GBM patients from CGGA database (mRNAseq_325) were defined as validation cohorts. Fragments Per Kilobase Million (FPKM) values were transformed to Transcripts Per Kilobase Million (TPM), and log_2_ (TPM+1) transformation was applied for the following analyses. LGG or GBM patients in the above cohorts were categorized into low- and high- expression groups according to their median expression of PLEKHA4. The demographic and clinical characteristics of enrolled patients with low and high PLEKHA4 expression are shown in **Supplementary Tables 1-6**.

### PLEKHA4 expression analysis

Tumor Immune Estimation Resource (TIMER) database (http://cistrome.shinyapps.io/timer/) was utilized to explore the expression profiling of PLEKHA4 between tumor and adjacent normal tissues. As for pan-cancer analysis integrating TCGA and GTEx, all of the expression data were normalized by converting them into log2(TPM+1) format. The PLEKHA4 expression was compared between tumor and normal samples using R software (version 3.6.3). Details of the cancer and matched normal tissue samples from TCGA and GTEx were listed in **Supplementary Table 7**. Protein expression analysis of PLEKHA4 was acquired using UALCAN database (http://ualcan.path.uab.edu) (35). Gene Expression Profiling Interactive Analysis database (GEPIA, http://gepia2.cancer-pku.cn/#index) was implemented to evaluate the expression of PLEKHA4 in LGG and GBM, as well as in different LGG and GBM subtypes (TCGA data). Representative immunohistochemistry (IHC) staining images were obtained from the Human Protein Atlas (HPA) database (http://www.proteinatlas.org/). The “pROC” and “ggplot2” packages were used to construct the receiver operating characteristic (ROC) curve to illustrate the prediction efficacy of PLEKHA4. Additionally, clinical-pathologic correlation analysis was conducted by linking PLEKHA4 expression data with pathological characteristics.

### Survival analysis

The glioma tissue microarray (ZL-BraG Sur1801) with detailed survival information of enrolled patients (**Supplementary Table 8**) was obtained from Shanghai Wellbio Biotech Co., Ltd (Shanghai, China). The LGG and GBM patients in TCGA and CGGA datasets, as well as the clinical samples included in the tissue microarray, were divided into high- and low- expression groups by median value of PLEKHA4, respectively. Overall survival (OS) and disease specific survival (DSS) analysis were performed using R software. Hazard ratios (HR) with 95% confidence intervals (CI) and log-rank *P*-value were determined using the R package “survival”. The “survminer” package was applied for visualization.

### Univariate and multivariate cox regression analysis

Univariate and multivariate Cox analysis were utilized to investigate the prognostic potential of PLEKHA4, WHO grade, age, gender, histological type, isocitrate dehydrogenase (IDH) status, 1p/19q codeletion, primary therapy outcome, radiation therapy and chemotherapy on OS or DSS in LGG. *P* value <0.05 was set as the cut-off criterion. Forest plots showing the HR, 95% CI and *P*-value were constructed using the R package “ggplot2”.

### Prognostic model generation and prediction

Based on the independent prognostic factors identified by multivariate Cox analysis, nomogram models were constructed to predict the 1-, 3-, and 5- year OS or DSS in patients with LGG using the “rms” and “survival” R packages. Calibration and DCA analysis were also performed to evaluate the accuracy and clinical applicability of the nomogram models using the “survival”, “rms” and “stdca.R” R packages.

### PLEKHA4 mutation profiles, methylation level and its prognosis

The copy number alteration (CNA) and mutation landscape of PLEKHA4 in LGG were investigated using the web-based platform cBioPortal (http://www.cbioportal.org). Shiny Methylation Analysis Resource Tool (SMART, http://www.bioinfo-zs.com/smartapp/) was used to analyze the pan-cancer methylation profiles of PLEKHA4, the correlation between CpG island methylation and PLEKHA4 expression, as well as the prognostic values of PLEKHA4 methylation in LGG (based on TCGA data). Integration and visualization of PLEKHA4 expression and DNA methylation in combination with the precise genomic location of the CpG sites were conducted using online tool MEXPRESS (https://mexpress.be/).

### GO, KEGG, and GSEA

Differentially expressed genes (DEGs) between high- and low- PLEKHA4 groups in TCGA-LGG cohort were identified using R package “DEseq2”. DEGs with a |log2Fold Change| >1.0 and adjusted *P*<0.05 were visualized by a volcano plot using “ggplot2” package. Meanwhile, the top 10 upregulated and downregulated DEGs were depicted by a heat map. Additionally, GO and KEGG enrichment analysis of DEGs were performed using “clusterProfiler” package. The top 10 enriched terms were visualized with the combination of gene expression data using “Goplot” and “ggplot2” packages (36). Meanwhile, GSEA analysis was performed using the “clusterProfiler” package with 1,000 permutations. Gene sets of “h.all.v7.2.symbols.gmt [Hallmarks]”, “c2.cp.v7.2.symbols.gmt [Curated]” and “c5.all.v7.2.symbols.gmt [Gene ontology]” in the MSigDB collections were chosen as the reference gene collections, respectively. The items with a false discovery rate (FDR) <0.25 and adjusted *P*<0.05 were considered as significant enrichment. “ggplot2” package was utilized for visualization.

### Identification of coexpression and interaction network

LinkedOmics (http://www.linkedomics.org/login.php) was utilized to screen genes coexpressed with PLEKHA4 using Spearman’s correlation test. Heatmaps of positively or negatively correlated genes with PLEKHA4 were acquired from the LinkFinder module. GSEA enrichment analysis was performed with the LinkInterpreter module. A PLEKHA4-related gene-gene interaction network was analyzed by GeneMANIA (http://www.genemania.org) online database (37). PLEKHA4 was also used to generate Protein-protein interaction (PPI) network using STRING (https://string-db.org/) database (38), and the minimum required interaction score was set at 0.4. The obtained PPI network was visualized by Cytoscape software.

### Immune infiltration analysis

Tumor purity was assessed using transcriptomic profiles of LGG cohorts from TCGA and CGGA with the ESTIMATE algorithm. Single sample Gene Set Enrichment Analysis (ssGSEA) was performed to analyze 28 immune signatures using the GSVA package. Heatmaps were plotted to display the association of PLEKHA4 expression with GSVA scores and ESTIMATE scores. ImmuneScore, StromalScore, and ESTIMATEScore were also compared between the low- and high- PLEKHA4 groups. Subsequently, the correlations between PLEKHA4 expression and the infiltration of major immune and stromal cells (including dendritic cells, B cells, neutrophils, macrophages, CAFs, etc.) in LGG were also confirmed using TIMER database. Moreover, the proportions of 22 tumor-infiltrating immune cells in LGG patients were calculated using the CIBERSORT algorithm. The CIBERSORT scores of immune cell subpopulations were compared between the low- and high- PLEKHA4 groups. *P* values were corrected for multiple comparisons using Bonferroni’s test, and a *P* value <0.05 was considered significant. An association between infiltrating M2 macrophages and cumulative survival was also investigated using CIBERSORT scores of M2 cells.

### Cell culture, siRNA transfection, and macrophage polarization

Human LN229 and THP-1 cells were maintained in DMEM or RPMI 1640 medium (HyClone, Logan, UT, USA) supplemented with 10% fetal bovine serum (FBS, Gibco, USA) and 1% penicillin and streptomycin (HyClone) at 37°C with 5% CO_2_. siRNAs targeting PLEKHA4 and negative control siRNAs were synthesized by Sangon Biotech (Shanghai, China). To induce PLEKHA4 silencing, siRNAs were transfected into LN229 cells for 48 h using Lipofectamine RNAiMAX (Thermo Fisher, Scientific, USA). The knockdown efficiency was then verified using RT-qPCR method as described previously (39). To polarize M0 to M1 macrophages, 2.5×10^5^ THP-1 cells were seeded into 6-well plate and stimulated with 320 nM phorbol-12-myristate-13-acetate (PMA; Beyotime Biotechnology, China) at 37°C for 6 h. To polarize M2 macrophages, cells were further incubated with IL-4 (20 ng/ml; Beyotime) and IL-13 (20 ng/ml; Beyotime) for 72 h at 37°C (24, 40). Identification of THP-1 derived M2 macrophages was performed using immunofluorescence (IF) staining for CD163 (ab182422, Abcam; 1:1500 dilution), as reported earlier (39).

### M2 macrophage infiltration assay

To examine the effect of PLEKHA4 on M2 macrophage infiltration, 2.5×10^5^ M2 macrophages were suspended in 300 μl of serum-free medium and seeded into the upper chambers of a transwell plate (8.0 μm pores, Corning, USA). 2.5×10^5^ LN229 cells were incubated with 700 μl of complete medium in the bottom chambers. After co-incubation at 37°C for 24 h, cells that infiltrated into the lower surface of the membrane were fixed with 500 μl methanol and stained with 1% crystal violet. Then, cells that on the upper surface of the membrane were wiped off using fluffy swabs, and the numbers of infiltrated M2 macrophages were counted manually in 6-8 randomly selected fields under the Olympus microscope (CKX53, Olympus, Japan). Six biological replicates were performed to determine the infiltration of M2 macrophages.

### Immunohistochemistry -based tissue microarrays

The glioma tissue microarray HBra-Gli060PG-01 was obtained from Shanghai Outdo Biotech Co., Ltd (Shanghai, China). Basic clinicopathologic information of enrolled patients was listed in **Supplementary Table 9**. IHC staining was carried out as mentioned elsewhere (41), using glioma tissue microarray HBra-Gli060PG-01 or ZL-BraG Sur1801. In brief, the tissue microarrays were deparaffinized in xylene and rehydrated using graded alcohol after heating at 63°C for 1 h. Antigen retrieval was then performed on the automatic IHC pretreatment system (PT Link, Dako North America, Inc., USA). Subsequently, the tissue microarrays were incubated with antibodies against PLEKHA4 (sc-376408, Santa Cruz Biotech, USA; 1:50 dilution), PD-L1 (ab210931, Abcam, USA; 1:3000 dilution), AIF1 (ab178846, Abcam; 1:1500 dilution), CD163 (ab182422, Abcam; 1:1700 dilution), NOS2 (18985-1-AP, ProteinTech Group, USA; 1:2000 dilution), and GADD45A (ab203090, Abcam; 1:1200 dilution), at 4°C overnight, respectively. After 3 washes with PBST, specimens were further placed into automatic IHC staining system (Autostainer Link 48, Dako) for blocking, secondary antibody binding and DAB color reaction. Finally, the samples were counterstained with hematoxylin and imaged using a slide scanner (NanoZoomer S360, Hamamatsu Photonics).

Images of the specimens were evaluated by two independent pathologists blinded to the clinicopathologic information. The quantity of stained cells was categorized as 0 (≤10%), 1 (11%–25%), 2 (26–50%), 3 (51–75%), and 4 (>75%). IHC intensity was scored as 0 (negative), 1 (weak brown), 2 (moderate brown), and 3 (strong brown). And the final IHC score of individual samples was determined by multiplication of quantity scores and intensity scores.

### Immunotherapy response prediction and drug sensitivity analysis

To predict patient response to Immune-Checkpoint Blocker (ICB) treatment, the mRNA-seq data from TCGA-LGG and CGGA-LGG were analyzed using the TIDE algorithms (Tumor Immune Dysfunction and Exclusion; http://tide.dfci.harvard.edu/) (42). Correlation analysis of PLEKHA4 expression with drug sensitivities was conducted *via* CellMiner database (http://discover.nci.nih.gov/cellminer) (43). Data were processed and visualized using the “limma” and “ggpubr” packages.

### Statistical analysis

The Wilcoxon rank sum test was used to compare PLEKHA4 expression levels between tumor and normal samples, as well as the differences of immune infiltration between the low- and high- PLEKHA4 groups. The Wilcoxon rank sum test, Kruskal-Wallis’s test, Chi-Square test, Fisher’s exact test and Spearman’s correlation test were used to clarify the correlations between clinical-pathologic features and PLEKHA4 expression. Log-rank test was utilized to evaluate the survival differences between groups. Correlations between certain variables were assessed by Spearman’s or Pearson’s correlation tests, and displayed as lollipop charts or chord graphs, using “circlize” and “ggplot2” package. Differences were considered statistically significant at *< 0.05, **< 0.01, ***< 0.001, ****< 0.001.

## Results

### PLEKHA4 is dysregulated in cancers

**Figure 1:**
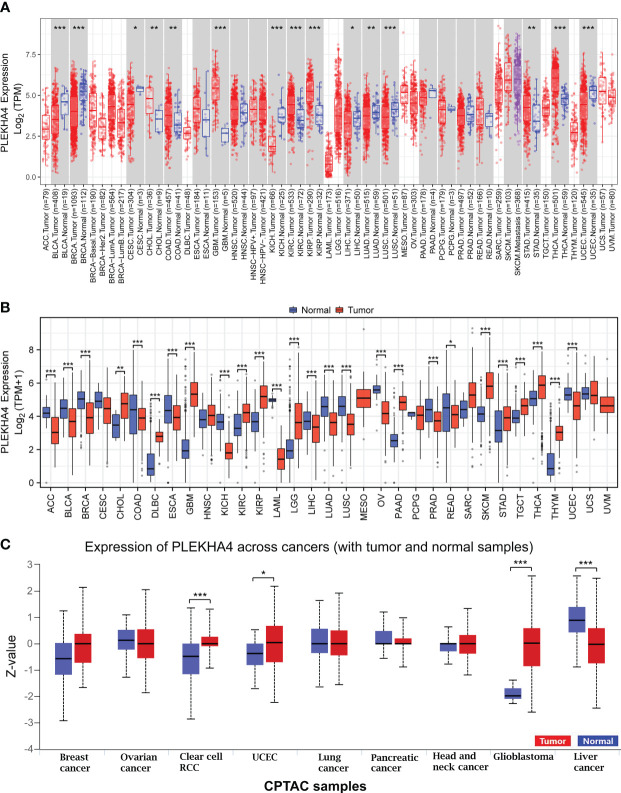
PLEKHA4 expression at pan-cancer level. **(A)** PLEKHA4 expression in different tumor types from TCGA database, as determined by the TIMER2.0. **(B)** Comparison of PLEKHA4 expression between tumor tissues from TCGA database and normal tissues from GTEx database. **(C)** Expression of the PLEKHA4 protein obtained from the large-scale proteome data available through UALCAN database. UALCAN projects used log-transformed expression values centered to standard deviations from the median within each cancer type. Z-values represent standard deviations from the median across samples for the given cancer type. *p < 0.05, **p < 0.01, ***p < 0.001.

To detect the expression profiles of PLEKHA4 in common human cancers, we evaluated the mRNA expression of PLEKHA4 in various tumor and normal tissues in TCGA dataset using TIMER2.0. As shown in **Figure 1A**, elevated PLEKHA4 expression was observed in various cancer types, including cholangio carcinoma (CHOL), colon adenocarcinoma (COAD), glioblastoma multiforme (GBM), kidney renal clear cell carcinoma (KIRC), kidney renal papillary cell carcinoma (KIRP), stomach adenocarcinoma (STAD), and thyroid carcinoma (THCA). On the contrary, PLEKHA4 was lower expressed in bladder urothelial carcinoma (BLCA), breast invasive carcinoma (BRCA), cervical and endocervical cancer (CESC), kidney chromophobe (KICH), liver hepatocellular carcinoma (LIHC), lung adenocarcinoma (LUAD), lung squamous cell carcinoma (LUSC), and Uterine Corpus Endometrial Carcinoma (UCEC).

The number size of normal tissues in the TCGA database is too small to be statistically convincing (e.g., the numbers of normal controls in GBM and LGG cohorts were 5 and 0, respectively), we therefore matched the normal tissues and cancer samples from GTEx and TCGA to reflect the PLEKHA4 expression in a more convincing manner. PCA analysis showed that samples from two databases tend to be clustered by sample types rather than by datasets, indicating that the sample type was the main factor causing the differences among samples (**Supplementary Figure 1**). Pan-cancer analysis were further carried out, and results showed that PLEKHA4 was dysregulated in majority of cancer types (**Figure 1B**). Specifically, PLEKHA4 expression was significantly elevated in CHOL, diffuse large B-cell lymphoma (DLBC), GBM, KIRC, KIRP, LGG, pancreatic adenocarcinoma (PAAD), skin cutaneous melanoma (SKCM), STAD, testicular germ cell tumors (TGCT), THCA, and thymoma (THYM). However, PLEKHA4 was down-regulated in adrenocortical carcinoma (ACC), BLCA, BRCA, colon adenocarcinoma (COAD), esophageal carcinoma (ESCA), KICH, acute myeloid leukemia (LAML), LIHC, LUAD, LUSC, ovarian serous cystadenocarcinoma (OV), prostate adenocarcinoma (PRAD) and UCEC, compared to GTEx normal controls.

Moreover, PLEKHA4 expression at a protein level was explored using the large-scale proteome data available through UALCAN. As displayed in **Figure 1C**, the protein expression of PLEKHA4 was strikingly elevated in glioblastoma samples (*P*<0.001), indicating that PLEKHA4 may play a vital role in the tumorigenesis of glioma.

### PLEKHA4 is upregulated in glioma

**Figure 2:**
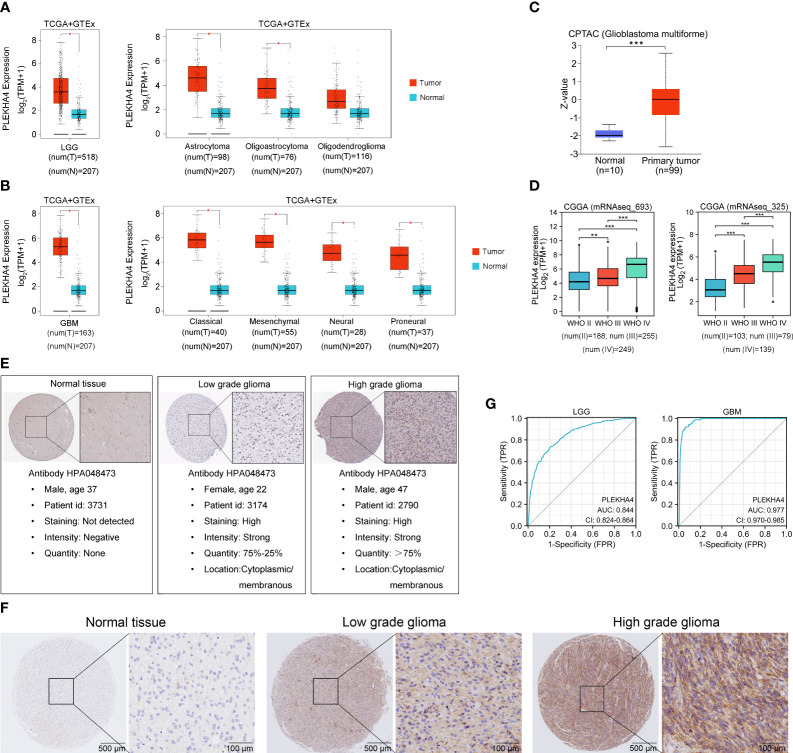
PLEKHA4 expression in glioma. **(A)** The expression of PLEKHA4 between normal and LGG tissues (left panel), as well as LGG tissues of different histological types (right panel), from GEPIA database. **(B)** The expression of PLEKHA4 between normal and GBM tissues (left panel), as well as GBM tissues of different histological types (right panel), from GEPIA database. **(C)** Protein levels of PLEKHA4 in glioblastoma obtained from UALCAN database. Z-values represent standard deviations from the median across samples for the given group. Log2 spectral count ratio values from UALCAN were first normalized within each sample profile, then normalized across samples. **(D)** The expression of PLEKHA4 in glioma tissues from CGGA database. **(E)** Protein levels of PLEKHA4 visualized by IHC *via* the HPA database. **(F)** IHC staining for PLEKHA4 in glioma and normal brain tissues provided by glioma tissue microarrays (ZL-BraG Sur1801). Panorama: scale bar = 500 μm; Enlarged: scale bar = 100 μm. **(G)** ROC curves showing the predictive performance of PLEKHA4 for discriminating the LGG (left panel) or GBM (right panel) from normal. *p < 0.05, **p < 0.01, ***p < 0.001.

To further confirm PLEKHA4 expression in glioma, GEPIA2 and UALCAN datasets were utilized to analyze PLEKHA4 expression at transcriptional and protein levels. As shown in **Figures 2A, B**, total LGG or GBM patients (left panel), as well as patients with different histological types (right panel), all displayed elevated PLEKHA4 expression than those in normal tissues. PLEKHA4 at protein level was also greatly upregulated in glioblastoma as compared with normal tissues (**Figure 2C**). To avoid the possible bias caused by a single database, PLEKHA4 expression was further examined using two independent CGGA datasets (mRNAseq_693 and mRNAseq_325), and results showed that PLEKHA4 was significantly upregulated in higher grade of gliomas (**Figure 2D**). Meanwhile, IHC results provided by the HPA database also displayed that PLEKHA4 protein, which was mainly distributed in the cytoplasm and membrane, was obviously elevated in both low grade and high-grade gliomas, compared with normal brain tissue (**Figure 2E**). This result was further validated using glioma and normal brain tissues provided by glioma tissue microarrays (**Figure 2F**). Furthermore, to determine the predictive power of PLEKHA4, ROC curves were constructed and the AUCs for LGG and GBM were 0.844 (95% CI=0.824-0.864) and 0.977 (95% CI=0.970-0.985), respectively (**Figure 2G**), suggesting that PLEKHA4 is an effective index for the diagnosis of LGG or GBM patients.

### High PLEKHA4 expression predicts poor prognosis in LGG

**Figure 3:**
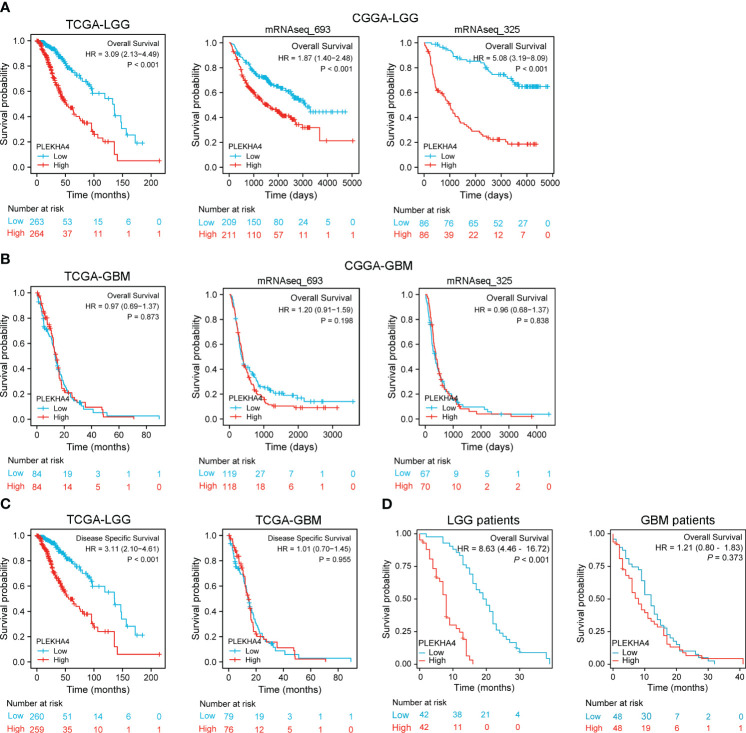
Prognostic potential of PLEKHA4 in glioma. **(A)** Kaplan‐Meier survival analysis of OS in TCGA-LGG and CGGA-LGG cohorts. **(B)** Kaplan‐Meier survival analysis of OS in TCGA-GBM and CGGA-GBM cohorts. **(C)** Survival curves of DSS from TCGA-LGG and TCGA-GBM cohorts. **(D)** Overall survival rate of PLEKHA4 in LGG (n = 84) and GBM patients (n = 96).

Kaplan-Meier survival analysis was conducted to assess the prognostic potential of PLEKHA4 in LGG and GBM. As shown in **Figure 3A**, patients with higher PLEKHA4 expression achieved poor OS prognosis in the TCGA-LGG cohort (HR=3.09, 95% CI=2.13-4.49, *P*<0.001), CGGA-LGG (mRNAseq_693) cohort (HR=1.87, 95% CI=1.40-2.48, *P*<0.001), and CGGA-LGG (mRNAseq_325) cohort (HR=5.08, 95% CI=3.19-8.09, *P*<0.001). In contrast, no obvious correlations were revealed between PLEKHA4 expression and OS in all the tested GBM cohorts (**Figure 3B**). Meanwhile, as shown in **Figure 3C**, high- PLEKHA4 group also achieved relatively worse DSS in LGG (HR=3.11, 95% CI=2.10-4.61, *P*<0.001), but not in GBM (HR=1.10, 95% CI=0.70-1.45, *P*=0.955). Moreover, the association between PLEKHA4 and prognosis was also validated using clinical samples of LGG (n=84) and GBM (n=96), and results clearly showed that a high PLEKHA4 expression was significantly related to adverse prognosis of LGG patients (HR=8.63, 95% CI=4.46-16.72, *P*<0.001), but not GBM (HR=1.21, 95% CI=0.08-1.83, *P*=0.373, **Figures 3D**).

### Increased PLEKHA4 expression is correlated with malignant phenotypes in LGG

**Figure 4:**
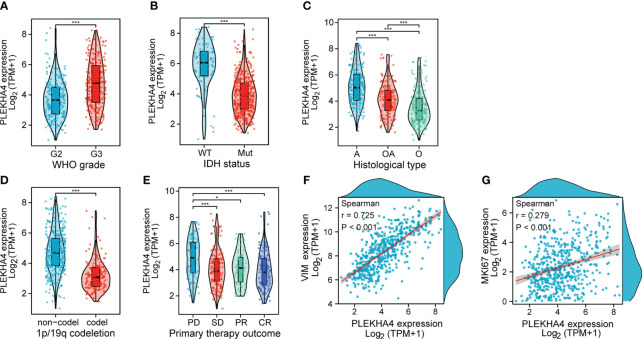
Associations between PLEKHA4 expression and different clinical characteristics in TCGA-LGG cohort. **(A)** WHO grade. **(B)** IDH status. **(C)** Histological type. **(D)** 1p/19q codeletion. **(E)** Primary therapy outcome. **(F, G)** Scatterplots showing the expression correlations of PLEKHA4 with **(F)** VIM or **(G)** MKI67. WT, IDH wild-type; Mut, IDH mutant; A, Astrocytoma; OA, Oligoastrocytomas; A, Astrocytoma; PD, Progressive Disease; SD, Stable Disease; PR, Partial Response; CR, Complete Response. *p < 0.05, ***p < 0.001.

To clarify the correlations between PLEKHA4 expression and malignant phenotypes of LGG, samples from TCGA and CGGA cohorts were stratified into subgroups by WHO grade, IDH status, histological type, 1p/19q codeletion, gender and progression status. Differential mRNA levels of PLEKHA4 were compared in different subgroups. As shown in **Figure 4**, higher PLEKHA4 expression was displayed in subgroups of WHO G3 (*P*<0.001), IDH wild-type (*P*<0.001) and 1p/19q non-codeletion (*P*<0.001), in which LGG patients tended to be more malignant. Meanwhile, PLEKHA4 expression was significantly different among subgroups stratified by histological type (*P*<0.001) and primary therapy outcome (*P*<0.001), and patients of astrocytoma (A) or progressive disease (PD) types usually have higher PLEKHA4 expression levels. Moreover, the correlations of PLEKHA4 with MKI67 (proliferation index) or VIM (invasion index) were also investigated, and results showed that PLEKHA4 expression correlated weakly with MKI67 (r=0.279, *P*<0.001), but strongly with VIM (r=0.725, *P*<0.001). These results were further verified using CGGA-LGG cohorts of mRNAseq_693 and mRNAseq_325 (**Supplementary Figure 2**), confirming the synchronization of PLEKHA4 overexpression and the malignant clinicopathological characteristics of LGG patients.

### PLEKHA4 shows good performance in predicting clinical prognosis

**Figure 5:**
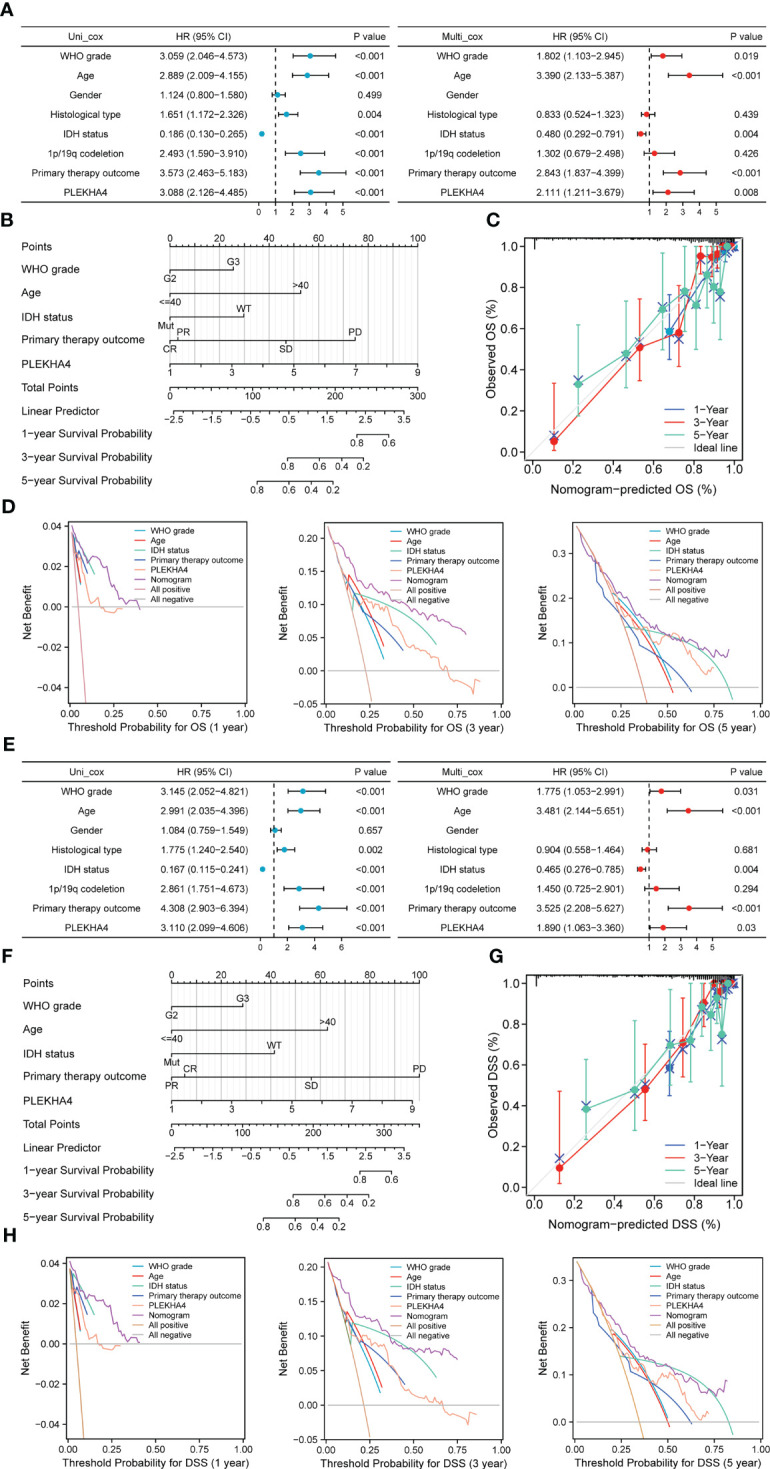
Prognostic value of PLEKHA4 in LGG. **(A, E)** Univariate and multivariate Cox regression analyses of PLEKHA4 expression and other clinical pathological factors for **(A)** OS and **(E)** DSS in the TCGA-LGG cohort. **(B, F)** Nomograms were constructed with PLEKHA4 expression and clinicopathologic variables to estimate 1-, 3- and 5- **(B)** OS and **(F)** DSS of LGG patients. **(C, G)** Calibration plots to verify the accuracy of the predicted 1-, 3- and 5- **(C)** OS and **(G)** DSS of LGG patients in the nomograms. **(D, H)** DCA curves to evaluate the accuracy and clinical applicability of the nomogram models for 1-, 3- and 5- **(D)** OS and **(H)** DSS of LGG patients. WHO grade, Age, IDH status, Primary therapy outcome and PLEKHA4 curves represent its own prognostic value. Nomogram curve represents the synthetical prognostic value of the abovementioned factors. All positive curve represents the theoretical best prognostic value, and all negative curve represents no prognostic value.

To investigate whether PLEKHA4 is an independent prognostic factor of LGG patients, Cox regression analysis was conducted using TCGA-LGG cohort. On univariate Cox regression analysis, the WHO grade (*P*<0.001), age (*P*<0.001), histological type (*P*=0.004), IDH status (*P*<0.001), 1p/19q codeletion (*P*<0.001), primary therapy outcome (*P*<0.001) and PLEKHA4 expression (*P*<0.001) were obviously correlated with the OS of LGG patients (**Figure 5A**, left panel). These factors were subjected to multivariate Cox regression analysis, and identified the WHO grade (*P*=0.019), age (*P*<0.001), IDH status (*P*=0.004), primary therapy outcome (*P*<0.001), and PLEKHA4 expression (*P*=0.008) as independent prognostic factors associated with OS (**Figure 5A**, right panel).

Next, to further evaluate the prognostic prediction ability of PLEKHA4 and its potential in clinical application, a nomogram model integrating the PLEKHA4 expression and other independent prognostic factors was constructed to predict the 1-, 3- and 5-year OS rates of LGG patients. The C- index value for the nomogram model was 0.858, indicating a moderate predictive accuracy for OS in LGG (**Figure 5B**). Calibration curves were plotted to compare the nomogram-predicted 1-, 3- and 5-year OS with actual OS rates. As shown in **Figure 5C**, the bias-corrected curves in the calibration plot conformed well to the ideal line (the 45° line), demonstrating an excellent predictive ability of the nomogram. Moreover, the DCA curves were also constructed to verify the clinical usefulness and reliability of this nomogram model, and results showed that our nomogram was superior to the WHO grade, age, IDH status, primary therapy outcome or PLEKHA4 expression level alone in predicting OS (**Figure 5D**). Meanwhile, Cox regression analysis and nomogram model were also validated using CGGA-LGG (mRNAseq_693) cohort (**Supplementary Figures 3A, B**). Calibration and DCA curves of all independent factors also proved the good performance of the diagnostic nomogram (**Supplementary Figures 3C, D**).

Considering that PLEKHA4 overexpression are also closely related to a poorer DSS in LGG, we next assessed the clinical performance of PLEKHA4 for predicting DSS in LGG patients. As expected, PLEKHA4 expression (*P*=0.03), as well as the WHO grade (*P*=0.031), age (*P*<0.001), IDH status (*P*=0.004) and primary therapy outcome (*P*<0.001), were identified as independent prognostic factors of DSS (**Figure 5E**). Nomogram for 1-, 3- and 5-year DSS rates was also constructed with the incorporation of these independent prognostic factors, and the C-index for DSS prediction was 0.872 (**Figure 5F**). The calibration curves for the nomogram-predicted DSS at 1-, 3- and 5-year also showed optimal concordance with the actual outcomes (**Figure 5G**). Additionally, shown by DCA curves, the nomogram yielded modest additional net benefit for 1- or 3-year DSS probability from other clinical factors, illustrating that our nomogram had potential for clinical utility for estimating a patient’s DSS (**Figure 5H**).

### DNA methylation correlates with PLEKHA4 expression and indicates a better outcome in LGG

**Figure 6:**
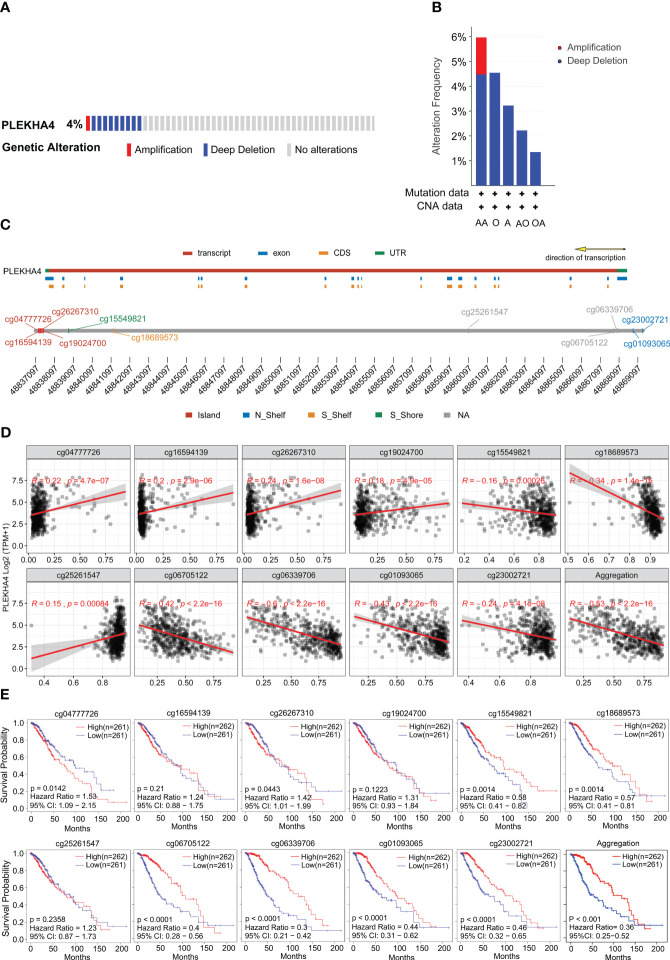
Genetic mutation and methylation alteration of PLEKHA4 in LGG. **(A)** PLEKHA4 mutation frequency in LGG obtained from the cBioPortal database. **(B)** Type and frequency of PLEKHA4 genomic alterations in LGG according to the cBioPortal database. AA, Anaplastic Astrocytoma; O, Oligodendroglioma; A, Astrocytoma; AO, Anaplastic Oligoastrocytoma; OA, oligoastrocytomas; **(C)** Detailed chromosomal distribution of the methylation probes associated with PLEKHA4 was obtained from SMART App. **(D)** Correlation analysis between the methylation status of CpG islands and PLEKHA4 expression in LGG by SMART App. **(E)** Prognostic value of PLEKHA4-specific CpG site methylation in LGG.

The genetic mutation of PLEKHA4 in LGG was analyzed with the TCGA data available at the cBioPortal database. In **Figures 6A, B**, the frequency of PLEKHA4 mutation in LGG was about 4%, with “deep deletion” as the prominent CNA (copy number alteration) type. Patients with the “amplification” of CNA usually had a higher level of PLEKHA4 expression. Whereas, the “amplification” of CNA was only found in LGG subtype of anaplastic astrocytoma (AA) with an extremely low frequency (~1.49%). This suggests that CNA may not be the main cause behind the high expression of PLEKHA4 in LGG patients.

Except for CNA, mRNA expression is also associated with the methylation level of genes. So, we further analyzed the status of PLEKHA4 methylation. As shown in **Supplementary Figure 4A**, PLEKHA4 gene in LGG was partially methylated, with a median β-value of 0.525. Genome-wide methylation mainly targeting CpG sites in gene expression regulatory elements, and a total of 11 CpG sites were found on PLEKHA4 gene. Detailed chromosomal distribution of the methylation probes associated with PLEKHA4 were shown in **Figure 6C** and **Supplementary Table 10**. The relationship between CpG methylation and PLEKHA4 expression was explored, and results showed that the overall methylation of CpG sites (Aggregation) negatively correlated with PLEKHA4 expression, with a correlation coefficient (R) of -0.53 (*P*<2.2e-16). With respect to CpG subtypes, methylation of cg06339706, cg01093065 and cg06705122 showed the strongest correlation with PLEKHA4 expression, and the correlation coefficients were -0.6 (*P*<2.2e-16), -0.43 (*P*<2.2e-16) and -0.42 (*P*<2.2e-16), respectively (**Figure 6D**). Furthermore, we systematically investigated the association of PLEKHA4 methylation and the clinical survival prognosis of LGG patients. Results showed that patients with low PLEKHA4 methylation had a worse survival probability than patients in the high group (**Figure 6E**, Aggregation). In terms of CpG subtypes, the methylations of cg06339706, cg01093065, cg06705122 and cg23002721 were highly associated with the adverse outcomes in LGG patients.

**Figure 7:**
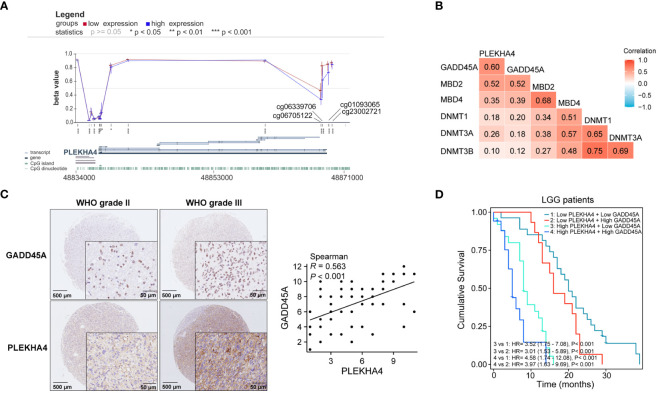
The correlation among CpG methylation, PLEKHA4 expression, and survival prognosis in LGG. **(A)** Visualization of PLEKHA4 expression and DNA methylation in combination with the precise genomic location of the CpG sites were obtained from the online tool MEXPRESS. **(B)** The heat maps showing the correlations of PLEKHA4 and DNA methylation/demethylation-related genes. **(C)** Representative images of IHC-based tissue microarrays showing the expression patterns of GADD45A and PLEKHA4 in LGG tissues (panorama: scale bar = 500 μm; enlarged: scale bar = 50 μm). The correlation coefficient between GADD45A and PLEKHA4 was assessed according to their IHC scores. **(D)** Survival analysis showing the prognostic significance of GADD45A and PLEKHA4 in clinical LGG samples (n=84). *p < 0.05, **p < 0.01, ***p < 0.001.

Meanwhile, visualization of PLEKHA4 expression, OS, and DNA methylation in combination with the precise genomic location of the CpG sites were obtained from the online tool MEXPRESS. The correlation among CpG methylation, PLEKHA4 expression, and survival prognosis in LGG were clearly shown on the integrated maps (**Supplementary Figure 4B**). It is worth noticing that, the methylation of cg23002721, cg01093065, cg06339706 and cg06705122, which were located in the promotor or near the transcription start site (TSS), was significantly down-regulated in high- PLEKHA4 group (**Figure 7A**). The correlation between DNA methylation/demethylation-related genes and PLEKHA4 was further analyzed. Results showed that PLEKHA4 expression was moderate correlated with DNA demethylation-related genes (GADD45A, MBD2, MBD4), but weakly correlated with transmethylases (DNMT1, DNMT3A, and DNMT3B) (**Figure 7B**), indicating that the dysregulation of CpG methylation on PLEKHA4 gene may be mediated by DNA demethylation. The expression correlation between PLEKHA4 and GADD45A were further verified using IHC based-tissue microarrays (r=0.563, *P*<0.001, **Figure 7C**). GADD45A-mediated CpG demethylation may be the main cause of PLEKHA4 upregulation in LGG. Moreover, the combined prognostic significance of PLEKHA4 and GADD45A was examined in clinical LGG samples (n=84). Survival curves clearly showed that LGG patients with high GADD45A and PLEKHA4 expression exhibited the worst cumulative survival (**Figure 7D**).

Furthermore, the prognostic potential of CpG methylation was evaluated. As shown in **Supplementary Figure 5**, AUC curves illustrated that the methylation of cg23002721, cg01093065, cg06339706 and cg06705122 were protective factors for 1-5 year- OS. Nomogram was constructed with PLEKHA4 expression and four CpG sites to estimate 1-, 3- and 5- OS of LGG patients, and the C-index for OS prediction was 0.806. Calibration curves proved the good performance of the diagnostic nomogram. Moreover, DCA curves were also plotted to verify the clinical reliability of the nomogram model, and results showed that the synthetical analysis of CpG methylation and PLEKHA4 (nomogram) usually achieved better net benefit than using methylation or PLEKHA4 expression alone, in predicting OS.

Collectively, the methylation correlates with the expression of PLEKHA4 and indicates a better outcome in LGG patients. The synthetical analysis of CpG methylation and PLEKHA4 expression can be more helpful for predicting the OS of LGG patients.

### PLEKHA4-related DEGs are highly enriched in immune processes

**Figure 8:**
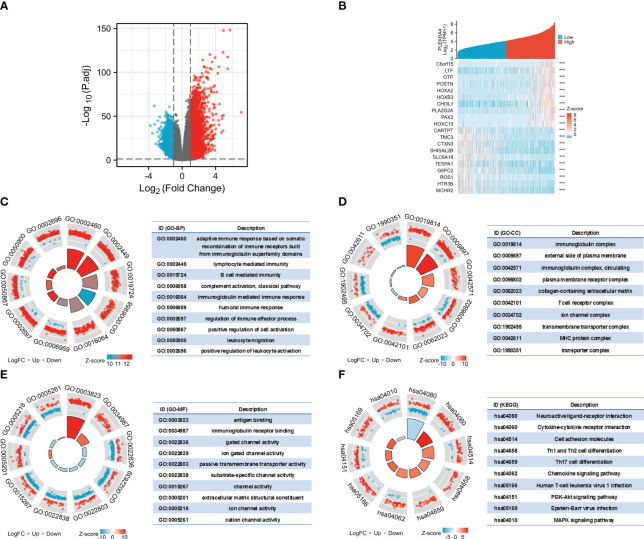
Functional enrichment analysis of DEGs between the PLEKHA4 high- and low-groups. **(A)** The volcano plot of 4169 DEGs. **(B)** Heatmaps showing the top 10 upregulated and downregulated DEGs. ***p < 0.001. **(C–E)** Top 10 enriched terms in the **(C)** BP, **(D)** CC and **(E)** MF based on GO analysis were shown as circle plots. **(F)** Circle plots of the top 10 enriched signaling pathways by KEGG enrichment analysis.

To interrogate the underlying effect of PLEKHA4 in LGG, DEGs between the PLEKHA4 high- and low- groups were analyzed using TCGA-LGG cohort. A total of 4169 DEGs were identified, of which 2767 genes were upregulated, and 1402 were downregulated (**Figure 8A**). The top 10 genes that were positively or negatively coexpressed with PLEKHA4 were shown in a heat map (**Figure 8B**). In the GO enrichment analysis, the top 5 enriched biological process (BP) terms were adaptive immune response based on somatic recombination of immune receptors built from immunoglobulin superfamily domains, lymphocyte mediated immunity, B cell mediated immunity, complement activation, immunoglobulin mediated immune response (**Figure 8C**). The top 5 enriched cellular component (CC) terms were immunoglobulin complex, external side of plasma membrane, immunoglobulin complex (circulating), plasma membrane receptor complex, collagen-containing extracellular matrix (**Figure 8D**). Among the molecular function (MF) terms, antigen binding, immunoglobulin receptor binding, and gated channel activity were significantly enriched (**Figure 8E**). Moreover, KEGG analysis indicated that neuroactive ligand-receptor interaction, cytokine-cytokine receptor interaction, cell adhesion molecules, Th1 and Th2 cell differentiation, Th17 cell differentiation, and chemokine signaling pathway were potential pathways in regulating the occurrence and development of LGG by PLEKHA4 (**Figure 8F**).

Additionally, GSEA were also implemented to identify the key pathways related to PLEKHA4. Among the GO, KEGG and HALLMARK terms of GSEA, most signaling pathways affected by PLEKHA4 were related to immune response and remodeling of TME, including immunoglobulin complex, B cell mediated immunity, antigen binding, complement activation, cytokine-cytokine receptor interaction, primary immunodeficiency, antigen processing and presentation, natural killer cell mediated cytotoxicity, interferon gamma response, interferon alpha response, and inflammatory response (**Supplementary Figures 6, 7**).

Taken together, these data highlight the close relationship between PLEKHA4 and immune cell infiltration, which may affect TME and induce LGG heterogeneity.

### PLEKHA4 are involved in the tumor-immune system interactions

To further explore the potential functions of PLEKHA4 in LGG, LinkedOmics database was used to study the coexpression patterns of PLEKHA4 screened from the TCGA-LGG cohort. The PLEKHA4-related genes were shown in a volcano map (**Supplementary Figure 8A**). The top 50 genes positively or negatively correlated with PLEKHA4 were selected and displayed in the heatmaps (**Supplementary Figure 8B**). Furthermore, GSEA enrichment analysis revealed that the coexpression genes of PLEKHA4 mainly participated in adaptive immune response, lymphocyte mediated immunity, regulation of immune effector process, type I interferon production, humoral immune response, etc. Reactome pathway analysis of GSEA indicated that these genes were positively related to antigen processing-cross presentation, interferon gamma signaling, interferon alpha/beta signaling, interleukin-10 signaling, etc. (**Supplementary Figure 8C**).

A PPI (protein-protein interaction) network was further constructed using STRING database and Cytoscape. The 5 nodes with the highest degree centrality were DNM2, MYO1C, TPM2, FLNC, MYL9 (**Supplementary Figure 9A**). Moreover, a gene-gene interaction network was obtained from the GeneMANIA database. Among the 20 genes associated with PLEKHA4, KLHL12, a regulator of Wnt signaling pathway and collagen export (30, 44), displayed greatest interacting frequency (**Supplementary Figure 9B**). Thereafter, KEGG enrichment analysis with respect to PLEKHA4-binding partners was carried out, and results showed that proteoglycans in cancer, leukocyte transendothelial migration, and synaptic vesicle cycle are among the top hits (**Supplementary Figure 9C**), further strengthened the potential of PLEKHA4 in modulating immune response and TME formation.

### PLEKHA4 correlated with tumor immunity in LGG

**Figure 9:**
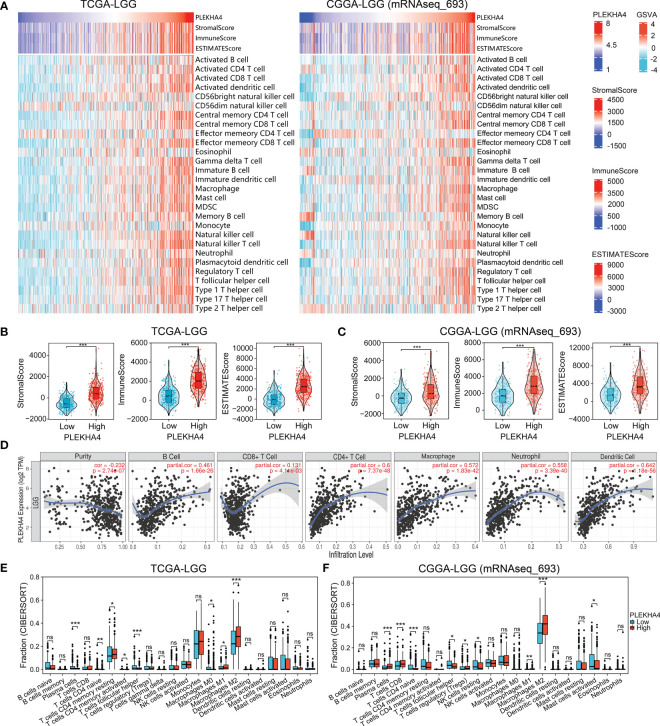
PLEKHA4 correlated with tumor immunity in LGG. **(A)** Heatmaps showing the association between PLEKHA4 expression and tumor purity (ESTIMATE algorithm), as well as the tumor-infiltrating immune cells (ssGSEA algorithm), in the TCGA-LGG and CGGA-LGG (mRNAseq_693) cohorts. **(B, C)** The comparison of StromalScore, ImmuneScore, and ESTIMATEScore between the high- and low- PLEKHA4 groups in the **(B)** TCGA-LGG and **(C)** CGGA-LGG (mRNAseq_693) cohorts. **(D)** Correlations between PLEKHA4 expression and the infiltration of B cells, CD8+ T cells, CD4+ T cells, macrophages, neutrophils, and DCs in LGG obtained from the TIMER database. **(E, F)** Comparison of tumor-infiltrating immune cells between the high- and low- PLEKHA4 groups in the **(E)** TCGA-LGG and **(F)** CGGA-LGG (mRNAseq_693) cohorts. ns (no significance), *p* ≥ 0.05, *p < 0.05, **p < 0.01, ***p < 0.001.

Considering that the abovementioned functional enrichment analyses all implied the participation of PLEKHA4 in tumor immune response, we next evaluated the association between PLEKHA4 expression and tumor purity using ESTIMATE algorithm. Specifically, the ImmuneScore, StromalScore, and ESTIMATEScore were positively correlated with PLEKHA4 mRNA expression in both TCGA-LGG and CGGA-LGG (mRNAseq_693) cohorts (**Figure 9A**). Statistically, the PLEKHA4-high group achieved significantly higher ImmuneScore, StromalScore, and ESTIMATEScore (**Figures 9B, C**). Similar results were also acquired in CGGA-LGG (mRNAseq_325) cohort (**Supplementary Figures 10A, B**), implying that samples in the high PLEKHA4 group contained greater tumor-infiltrating immune cells and stromal cells.

ssGSEA were further applied to analyze the correlation between PLEKHA4 expression and tumor-infiltrating immune cells. In the heatmaps of immune landscape (**Figure 9A**, **Supplementary Figure 10A**), PLEKHA4 expression presented strongly positive correlations with most immune cells in the tested three cohorts, including the macrophages, activated dendritic cells (DCs), central memory CD4 T cells, etc. To clarify the specific cell types modulated by PLEKHA4, the correlations between PLEKHA4 and diverse sets of immunological markers were assessed. As listed in **Tables 1**, **2** and **Supplementary Table 11**, PLEKHA4 expression was remarkably associated with DC markers (HLA-DPB1, HLA-DRA, etc.), M2 macrophage markers (VSIG4, TGFB1), monocyte markers (CD86, CD115), TAM markers (CCL2, CD68, etc.), neutrophil markers (CD11b, FCGR3B), Th1 markers (T-bet, STAT1), Th2 markers (STAT5A, IL6, GATA3), exhausted T cell markers (PD-1, TIM-3), etc. Besides, TIMER software was adopted to evaluated the immune infiltration status of LGG patients. **Figure 9D** showed that PLEKHA4 expression was strongly correlated with the infiltration of DCs (r=0.642, *P*=1.18e-56), CD4+ T cells (r=0.6, *P*=7.37e-48), macrophages (r=0.572, *P*=1.83e-42) and neutrophils (r=0.558, *P*=3.39e-40), and moderately with B cells (r=0.461, *P*=1.66e-26). However, PLEKHA4 was very weakly correlated with the infiltration of CD8+ T cells (r=0.131, *P*=4.11e-03). These findings further confirmed the robust interaction between PLEKHA4 and immune cells infiltration.

**Table 1:** Correlation analysis between PLEKHA4 and various gene markers of immune cells in the TCGA-LGG cohort.

Description	Gene markers	Cor	*P*
CD8+ T cell	CD8A	0.23	***
	CD8B	0.14	***
T cell (general)	CD3D	0.48	***
	CD3E	0.51	***
	CD2	0.51	***
	CD3G	0.36	***
	CD4	0.61	***
B cell	CD19	0.50	***
	CD79A	0.34	***
	CD79B	0.46	***
	MS4A1	0.16	***
Monocyte	CD86	0.59	***
	CD115 (CSF1R)	0.48	***
TAM	CCL2	0.48	***
	CD68	0.62	***
	IL10	0.47	***
M1 Macrophage	TNOS (NOS2)	−0.14	***
	PTGS2	0.12	**
M2 Macrophage	CD163	0.49	***
	VSIG4	0.52	***
	TGFB1	0.67	***
Neutrophils	CD11b (ITGAM)	0.54	***
	CCR7	0.26	***
	FCGR3B	0.40	***
	CXCR2	0.39	***
NK cell	KLRF1	0.09	*
	GNLY	0.43	***
	NKG7	0.44	***
	KLRD1	0.27	***
Dendritic cell	HLA-DPB1	0.65	***
	HLA-DQB1	0.46	***
	HLA-DRA	0.66	***
	HLA-DPA1	0.63	***
	CD11C (ITGAX)	0.53	***
Th1	T-bet (TBX21)	0.39	***
	STAT1	0.40	***
Th2	GATA3	0.45	***
	STAT6	0.36	***
	STAT5A	0.62	***
	IL6	0.41	***
Tfh	BCL6	0.05	0.25
	CXCR5	0.07	0.11
Th17	STAT3	0.41	***
	IL17A	0.053	0.23
Treg	FOXP3	−0.17	***
	STAT5B	−0.08	0.07
Tex	PD-1 (PDCD1)	0.47	***
	CTLA4	0.36	***
	LAG3	0.32	***
	TIM-3 (HAVCR2)	0.64	***
Mast cells	TPSB2	−0.14	**
	TPSAB1	0.02	0.58
	HDC	0.18	***

TAM, tumor-associated macrophage; Th, T helper cell; Tfh, Follicular helper T cell; Treg regulatory T cell, Tex, exhausted T cell; Cor, R value of Spearman’s correlation. *< 0.05, **< 0.01, ***< 0.001.

**Table 2:** Correlation analysis between PLEKHA4 and various gene markers of immune cells in the CGGA-LGG (mRNAseq_693) cohort.

Description	Gene markers	Cor	*P*
CD8+ T cell	CD8A	0.35	***
	CD8B	0.46	***
T cell (general)	CD3D	0.55	***
	CD3E	0.58	***
	CD2	0.56	***
	CD3G	0.45	***
	CD4	0.51	***
B cell	CD19	0.45	***
	CD79A	0.53	***
	CD79B	0.50	***
	MS4A1	0.22	***
Monocyte	CD86	0.48	***
	CD115 (CSF1R)	0.39	***
TAM	CCL2	0.41	***
	CD68	0.58	***
	IL10	0.36	***
M1 Macrophage	TNOS (NOS2)	0.03	**
	PTGS2	-0.05	**
M2 Macrophage	CD163	0.57	***
	VSIG4	0.52	***
	TGFB1	0.69	***
Neutrophils	CD11b (ITGAM)	0.47	***
	CCR7	0.31	***
	FCGR3B	0.11	*
	CXCR2	0.25	***
NK cell	KLRF1	0.27	***
	GNLY	0.48	***
	NKG7	0.56	***
	KLRD1	0.31	***
Dendritic cell	HLA-DPB1	0.52	***
	HLA-DQB1	0.36	***
	HLA-DRA	0.61	***
	HLA-DPA1	0.60	***
	CD11C (ITGAX)	0.40	***
Th1	T-bet (TBX21)	0.54	***
	STAT1	0.40	***
Th2	GATA3	0.47	***
	STAT6	0.35	***
	STAT5A	0.59	***
	IL6	0.42	***
Tfh	BCL6	0.29	***
	CXCR5	0.32	***
Th17	STAT3	0.38	***
	IL17A	0.41	***
Treg	FOXP3	0.30	***
	STAT5B	0.20	***
Tex	PD-1 (PDCD1)	0.56	***
	CTLA4	0.33	***
	LAG3	0.52	***
	TIM-3 (HAVCR2)	0.48	***
Mast cells	TPSB2	0.36	***
	TPSAB1	0.39	***
	HDC	0.33	***

TAM, tumor-associated macrophage; Th, T helper cell; Tfh, Follicular helper T cell; Treg, regulatory T cell; Tex, exhausted T cell; Cor, R value of Spearman’s correlation. *< 0.05, **< 0.01, ***< 0.001.

Furthermore, CIBERSORT algorithm was employed to calculate the proportions of tumor-infiltrating immune cells in samples of TCGA-LGG and CGGA-LGG cohorts. Data were compared between the PLEKHA4 high- and low- groups. As shown in **Figures 9E, F** and **Supplementary Figure 10C**, M2 macrophages, M1 macrophages, plasma cells and T cells CD4 naive were the main immune cells affected by PLEKHA4 expression. Among them, M1 and M2 macrophages were apparently increased, but T cells CD4 naive were decreased in the PLEKHA4-high group than those in the PLEKHA4-low group. It is worth noticing that, in the PLEKHA4-high groups of the tested TCGA-LGG, CGGA-LGG (mRNAseq_693) and CGGA-LGG (mRNAseq_325) cohorts, the proportions of M2 macrophages were strikingly higher than others, with the fractions up to (28.3 ± 12.5) %, (41.3 ± 13.7) % and (47.5 ± 13.5) %, respectively, inferring that PLEKHA4 might play a vital role in regulating M2 macrophage infiltration in LGG.

### PLEKHA4 regulates M2 macrophage polarization in LGG and enhances the infiltration of M2 macrophages *in vitro*

**Figure 10:**
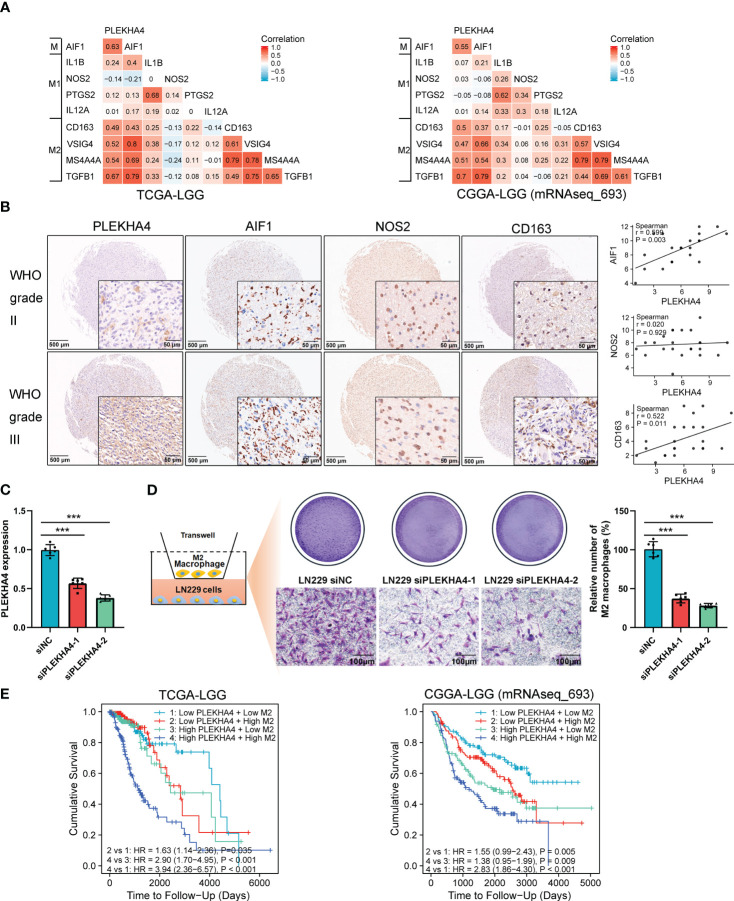
PLEKHA4 regulates the polarization and infiltration of M2 Macrophages in LGG. **(A)** The heat maps showing the correlations of PLEKHA4 and phenotype markers of monocytes (AIF1), M1 macrophages (IL1B, NOS2, PTGS2, IL12A), and M2 macrophages (CD163, VSIG4, TGFB1, MS4A4A) in the TCGA-LGG and CGGA-LGG (mRNAseq_693) cohorts. **(B)** PLEKHA4, AIF1, NOS2 and CD163 expression levels in LGG as determined by IHC-based tissue microarrays. Representative images were shown (panorama: scale bar = 500 μm; enlarged: scale bar = 50 μm). Scatterplots described the correlation coefficients of PLEKHA4 with AIF1, NOS2, or CD163. **(C)** Analysis of PLEKHA4 expression in LN229 cells transfected with siNC or siRNAs targeting PLEKHA4. ***p < 0.001. **(D)** Infiltration of THP-1-derived M2 macrophages in LN229 cells with siNC or siPLEKHA4 transfection. ***p < 0.001. **(E)** Kaplan‐Meier survival analysis showing the prognostic significance of PLEKHA4 based on M2 macrophage infiltration in the TCGA-LGG and CGGA-LGG (mRNAseq_693) cohorts.

Previous studies have shown that macrophage polarization from the type M1 to M2 promotes tumorigenesis in various cancers (45). To clarify the mechanism by which PLEKHA4 regulating M2 macrophage infiltration in LGG, the relationship between the expression of PLEKHA4 and classical phenotype markers of monocyte, M1, and M2 macrophage were investigated using the correlation coefficient analysis. Interestingly, we found that PLEKHA4 had strongly positive correlation with monocyte marker (AIF1) and M2 macrophage markers (CD163, VSIG4, TGFB1, MS4A4A), but very weakly correlated with M1 macrophage markers (IL1B, NOS2, PTGS2, IL12A) in TCGA-LGG, CGGA-LGG (mRNAseq_693) and CGGA-LGG (mRNAseq_325) cohorts (**Figure 10A**, **Supplementary Figure 11A**). The expression correlations between PLEKHA4 and those markers were further verified using TIMER database. As expected, PLEKHA4 were significantly correlated with M2 macrophage markers TGFB1 (r=0.614, *P*=7.78e-51) and MS4A4A (r=0.496, *P*=5.07e-31), whereas the correlation coefficients were extremely low with M1 macrophage markers NOS2 (r=-0.162, *P*=3.79e-04) and PTGS2 (r=0.073, *P*=1.13e-01) (**Supplementary Figures 11B, C**). Moreover, IHC based-tissue microarrays indicated that the intensity of AIF1 and CD163 was stronger in WHO grade III LGGs than that in WHO grade II. Spearman’s correlation analysis showed that PLEKHA4 expression was positively correlated with AIF1 (r=0.599, *P*=0.003) and CD163 (r=0.522, *P*=0.011). Whereas, NOS2 displayed no significant correlation with PLEKHA4 expression (**Figure 10B**). These findings further implicated that PLEKHA4 promoted the polarization of antitumor M1 to protumor M2 macrophages, which may be the leading mechanism by which PLEKHA4 regulating M2 macrophage infiltration in LGG.

To verify the effect of PLEKHA4 on M2 macrophage infiltration *in vitro*, THP-1 cells were differentiated into M2 macrophages according to the classical inducing methods (24, 40), and IF staining was employed to identify the THP-1 derived M2 macrophages. As shown in **Supplementary Figure 12**, more than 98% cells were strongly positive for CD163, indicating that the THP-1 cells have been successfully polarized into M2 macrophages. PLEKHA4 silencing was induced in LN229 cells using siRNAs targeting PLEKHA4 (**Figure 10C**). M2 macrophage infiltration assays showed that silencing of PLEKHA4 in LN229 cells remarkably reduced the infiltration of THP-1-derived M2 macrophages (**Figure 10D**).

Because elevated PLEKHA4 expression is correlated with unfavorable prognosis in LGG, and immune infiltration plays essential roles in patient outcomes (46), the prognostic significance of PLEKHA4 based on M2 macrophage infiltration was examined in TCGA-LGG and CGGA-LGG (mRNAseq_693) cohorts. Kaplan-Meier plotter results clearly showed that LGG patients with high PLEKHA4 expression and increased M2 macrophage infiltration exhibited the worst cumulative survival (**Figure 10E**). Meanwhile, in subgroups with low PLEKHA4 expression, LGG patients with higher M2 macrophage infiltration also displayed relatively poorer cumulative survival than those with lower M2 infiltration (**Figure 10E**). Similar results were also displayed in the CGGA-LGG (mRNAseq_325) cohort (**Supplementary Figure 11D**), indicating that the infiltration abundance of M2 macrophages was negatively correlated with the cumulative survival rate in LGG. Given that the PLEKHA4 expression is positively correlated with the infiltration level of M2 macrophages, our data indicate that PLEKHA4 may affect the LGG prognosis partially through intervening in the infiltration of M2 macrophages.

### PLEKHA4 reshapes the tumor microenvironment in LGG

**Figure 11:**
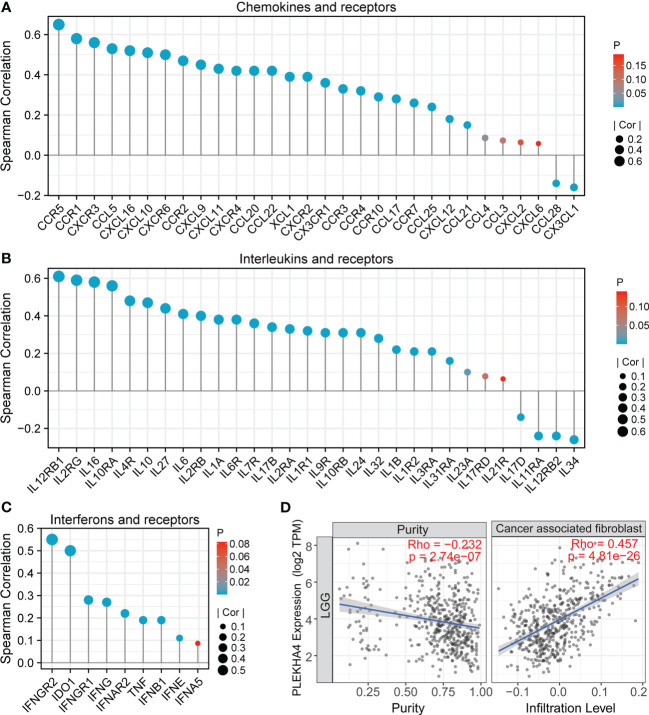
Association between PLEKHA4 expression and microenvironment of LGG. **(A-C)** Correlation analysis between PLEKHA4 and **(A)** cytokines, **(B)** interleukins or **(C)** interferons, as well as their corresponding receptors in the TCGA-LGG cohort. **(D)** Correlations between PLEKHA4 expression and the infiltration of cancer associated fibroblast by TIMER algorithm.

Except for tumor cells and infiltrating immune cells, TME also contains stromal cells and soluble factors that support tumor growth and progression. We next evaluated the correlation between PLEKHA4 expression with chemokines, interleukins, interferons and their receptors, as well as the infiltration of CAFs (a kind of stromal cells) in the TME of TCGA-LGG patients. In **Figures 11A-C**, PLEKHA4 expression was found to be significant correlated with chemokines (CCL5, CXCL16, CXCL10, CXCL9, CXCL11, etc.), interleukins (IL16, IL10, IL27, IL6, etc.), interferons (IFNG, etc.), and their corresponding receptors (CCR5, CCR1, CXCR3, CXCR6, IL12RB1, IL2RG, etc.), which have been found to direct immune cell infiltration and affect polarization state of macrophages (47–49). These results were further confirmed in the CGGA-LGG (mRNAseq_693) cohort (**Supplementary Figure 13**). Moreover, PLEKHA4 expression was closely related to the infiltration of the stromal CAFs (r=0.457, *P*=4.81e-26, **Figure 11D**), which can secret various cytokines, growth factors and chemokines, and consequently facilitate the recruitment of suppressive immune cells into the TME (50, 51). Taken together, our results indicate that PLEKHA4 may participate in the shaping of immunosuppressive TME in LGG patients.

### PLEKHA4 is a predictor for immunotherapy

**Figure 12:**
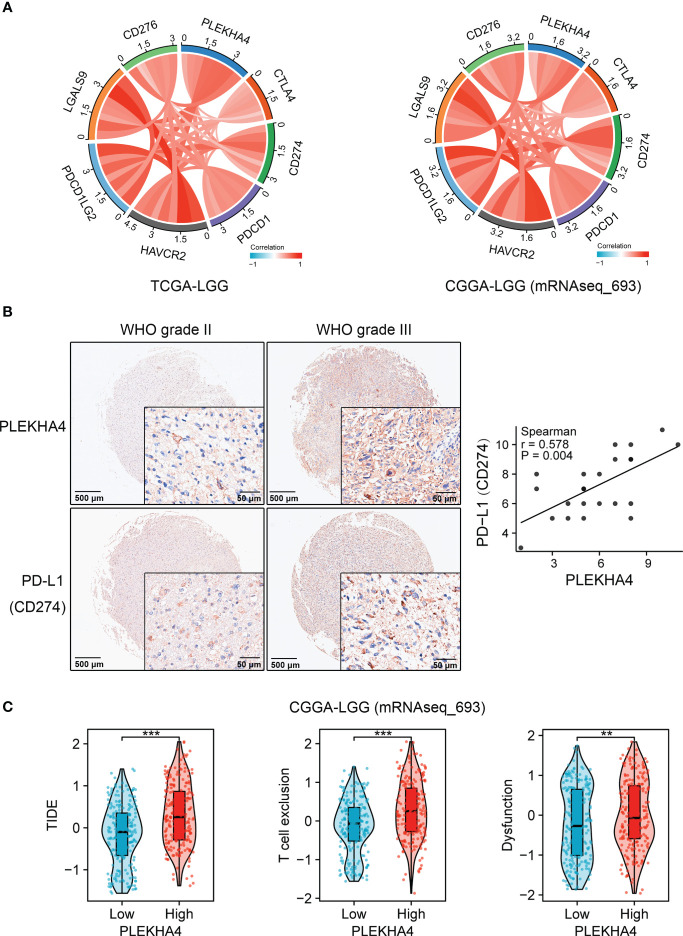
Clinical significance of PLEKHA4 in guiding immunotherapy. **(A)** Chord diagrams showing the correlations of PLEKHA4 and immune-related checkpoints in the TCGA-LGG and CGGA-LGG (mRNAseq_693) cohorts. **(B)** Representative images of IHC-based tissue microarrays showing the expression patterns of PLEKHA4 and PD-L1 in LGG tissues (panorama: scale bar = 500 μm; enlarged: scale bar = 50 μm). The correlation coefficient between PLEKHA4 and PD-L1 was assessed according to their IHC scores. **(C)** Comparison of TIDE, T cell exclusion and dysfunction scores between the high- and low- PLEKHA4 groups in the CGGA-LGG (mRNAseq_693) cohort. **p < 0.01, ***p < 0.001.

PLEKHA4 was manifested to substantially intervene in tumor immunity, prompting us to gain insight into its clinical significance in predicting immunotherapy response. Spearman’s correlation analysis showed that PLEKHA4 was strongly and significantly correlated with well-known immune checkpoints CTLA4, PD-L1 (CD274), PDCD1, HAVCR2, PDCD1LG2, CD276, etc. in the tested three cohorts (**Figure 12A**, **Supplementary Figure 14A**). Glioma tissue microarrays were adopted to verify the expression patterns of PLEKHA4 and PD-L1 in clinical LGG specimens. IHC staining demonstrated that PLEKHA4 expression was positively correlated with PD-L1 (r=0.578, *P* = 0.004, **Figure 12B**). TIDE algorithm was further used to predict the response of LGG patients to ICBs therapy (anti-PD-1 and anti-CTLA4) in CGGA-LGG (mRNAseq_693) cohort. Specific TIDE scores were listed in **Supplementary Table 12**. The results demonstrated that patients in the high-PLEKHA4 group achieved higher TIDE scores, as well as T cell exclusion scores and dysfunction scores (**Figure 12C**), indicating that patients with higher PLEKHA4 expression could achieve less sensitivity to ICBs treatment because of T cell exclusion and immune dysfunction. Similar results were acquired in CGGA-LGG (mRNAseq_325) dataset (**Supplementary Figure 14B, Table 13**). Taken together, these data demonstrated that patients with low PLEKHA4 expression may benefit from immunotherapy more clinically and PLEKHA4 can serve as an immunotherapy predictor for LGG patients.

### PLEKHA4 expression correlates with drug sensitivities

**Figure 13:**
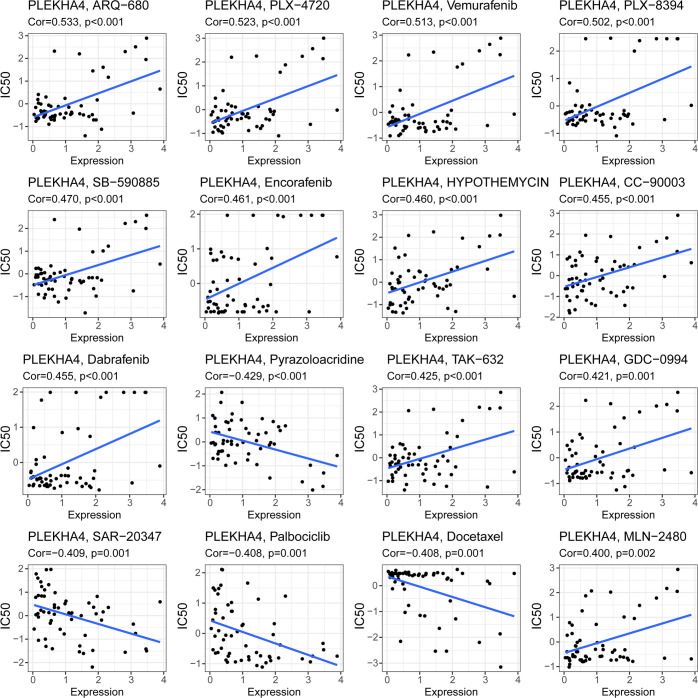
Correlation of PLEKHA4 expression with the sensitivities of anticancer drugs. The top 16 drugs with significant correlations with PLEKHA4 expression were showed.

CellMiner was utilized to explore the correlation between PLEKHA4 with drug response. The top 16 anticancer drugs that showed significant correlations with PLEKHA4 expression were depicted in **Figure 13**. The results suggested that high PLEKHA4 expression could increase the drug IC50s and decrease the drug sensitivities of B-Raf inhibitors, including PLX-4720, Vemurafenib, PLX-8394, SB-590885, Encorafenib, Dabrafenib, TAK-632 and MLN-2480, as well as the ERK and MEK inhibitors HYPOTHEMYCIN, GDC-0994 and CC-90003, indicating that PLEKHA4 expression might be related to the drug resistance of tumor cells. Whereas, PLEKHA4 was found to be significantly negatively correlated with IC50s of Pyrazoloacridine (inhibitor for topoisomerases 1 and 2), SAR-20347 (TYK2/JAK1/JAK2/JAK3 inhibitor), Palbociclib (selective CDK4/CDK6 inhibitor) and Docetaxel (microtubule depolymerization inhibitor), which provided guidance for the treatment of LGG patients with high PLEKHA4 expression.

## Discussion

Glioma, including LGG and GBM, is the most common type of primary intracranial tumors with poor prognosis and limited therapeutic options. Even though the clinical outcome of LGG is relatively better than GBM, LGG always invariably develops into secondary GBM (6–8). Many tumor-related events occur prior to reaching this stage, providing an optimal intervention window for glioma management. Therefore, an urgent strategy is now needed to explore the molecular mechanisms of LGG tumorigenesis and identify novel biomarkers to develop individual therapeutic strategies and new treatments to improve patient outcomes.

In this study, PLEKHA4 expression was found to be significantly upregulated in LGG and GBM at both transcriptional and protein levels, compared with the normal tissues. However, Kaplan–Meier survival analysis demonstrated that PLEKHA4 overexpression was significantly associated with OS and DSS of LGG patients, but not with those of GBM. Meanwhile, increased PLEKHA4 expression was associated with grade, IDH wildtype, 1p/19q non-codeletion, histological type, and VIM expression (marker of invasion) in LGG patients, further supporting the synchronization of PLEKHA4 overexpression and the malignant behaviors of LGG patients. What is more, Cox regression analysis identified that, except for WHO grade, age, IDH status and primary therapy outcome, PLEKHA4 expression was also a strong prognostic factor associated with both OS and DSS of LGG. Nomograms were further constructed combining the identified independent prognostic factors. Calibration and DCA curves proved that the nomograms could accurately predict the 1-, 3- and 5-year OS or DSS for patients with LGG. Moreover, the discrimination of our nomograms was confirmed higher than any single predictors, indicating the importance of an integrated predictive model.

DNA methylation is found to be strongly associated with gene expression regulation and therefore in the development of different pathologies (52). In the current study, the mechanism of elevated PLEKHA4 expression in LGG was investigated, and results showed that PLEKHA4 overexpression was negatively correlated with the methylation of cg06339706, cg01093065, cg06705122 and cg23002721, which were located in the promotor or near the TSS of PLEKHA4 gene. Meanwhile, the high-PLEKH4 expression group showed obviously decreased methylation in above-mentioned CpG sites. PLEKHA4 expression was significantly correlated with GADD45A which mediates DNA demethylation (53). Although many mechanisms can give rise to elevated gene expression, the demethylation of CpG cites is one of the main regulatory mechanisms underlying PLEKHA4 expression. What is more, DNA methylation is reported as the sole type of methylation that has been largely translated into clinics and used for, mostly, early diagnosis and prognosis (54, 55). Interestingly, we found that the methylation of the above CpG sites was adverse associated with the prognosis of LGG, which is consistent with the prognostic value of the mRNA expression of this gene. The synthetical analysis of CpG methylation and PLEKHA4 performed better than any single index in predicting OS of LGG patients, indicating that these CpG sites can aid LGG tumor progression monitoring and serve as prognostic markers as well, to identify patients with “high-risk”.

To further elucidate the potential role of PLEKHA4 in LGG progression, GO, KEGG and GSEA enrichment analysis were performed using DEGs. Many terms associated with immune response and TME remodeling were identified, including adaptive immune response, lymphocyte mediated immunity, complement activation, cytokine-cytokine receptor interaction, Th1 and Th2 cell differentiation, complement activation, etc. Meanwhile, we also manifested that the PLEKHA4 level could alter chemokine and interferon signaling pathway, as well as collagen-containing extracellular matrix, in LGG patients. TME in LGG patients is usually consisted of multiple components, including tumor cells, infiltrated immune cells, parenchyma cells, tumor-related soluble factors, ECM, etc. (2, 21, 22). Since the above analysis implied that the PLEKHA4 expression was associated with immune response, tumor-related soluble factors, and ECM in LGG, it inferred that PLEKHA4 plays a vital role in the tumor-immune system interactions and TME formation in LGG patients. Analyses of the PLEKHA4-binding partners and coexpression network further supported our findings. It is worth noticing that, KLHL12, the E3 ligase that modulates Wnt/β-catenin signaling pathway and collagen export (30, 44), is shown to be the key binding partner with PLEKHA4. Wnt/β-catenin signaling was reported to play a key role in reshaping the TME *via* fine crosstalk between transformed cells in the latest developments (56–58), implying that PLEKHA4 may exert its effect on immunoediting through interacting with KLHL12. Further investigation should be carried out to clarify the interactions between them.

To better understand the relationship between PLEKHA4 and tumor-immune system in LGG, we examined the role of PLEKHA4 in immune infiltration using the ESTIMATE, ssGSEA, as well as the TIMER algorithms. The results showed that PLEKHA4 expression was positively corelated with an increased infiltration of various immune cell types in LGG, including macrophages, CD4+ T cells, activated DCs, neutrophils, etc. The immune cells aggregate and are involved in the tumor immune network, which consequently facilitating immune evasion. Multiple studies have shown that DCs can present antigenic peptides on MHC molecules, thus activating CD4+ cells (59). Naive CD4+ T cells are then differentiated into a variety of effector subsets that present distinct immune functions. These subsets include Th1, Th2, Th17, T follicular helper, and regulatory T cell populations, etc. (60). Th1 cells, which produce IFN-γ and IL-2, evoke cell-mediated immunity (61, 62). Whereas, Th2 cells, which produce IL-4, IL-5, IL-6, IL-13, etc., prompt strong antibody responses, but inhibit several functions of phagocytic cells (61, 62). This is consistent with our functional enrichment results which showed that PLEKHA4 was correlated with the biological process of antigen processing and presentation, Th1 and Th2 cell differentiation, immunoglobulin mediated immune response, etc.

At present, infiltration of TAMs is considered to be one of the major causes of the immunosuppressive microenvironment in LGG (63). The M1 macrophages, occurring after toll-like receptor 4 (TLR4) ligands and/or IFN-γ exposure, is pro-inflammatory and anti-tumor (45, 63, 64). Conversely, the M2 macrophages, typically acquired after stimulation with IL-4, IL-10 and/or IL-13, is considered protumorigenic (45, 63, 64). Interestingly, calculations of the proportion of infiltrating immune cells by CIBERSORT revealed that the majority of TME-resident immune cells in LGG were M2 macrophages, with a substantial proportion up to 17-47%. Multiple studies have shown that M2 macrophages can secret high levels of IL-10, TGF-β, EGF, and low levels of IL-12, which overall promote invasion and angiogenesis of tumors (63, 64). More importantly, we further manifested that the number of M2 cells was significantly abundant in PLEKHA4-high group compared to the PLEKHA4-low group, implying that PLEKHA4 may manipulate the immunosuppressive TME in LGG through regulating the polarization and infiltration of M2 macrophages. This point was further strengthened by M2 macrophage infiltration assay *in vitro* using THP-1-derived M2 macrophages and LN229 cells with PLEKHA4 silencing. What is more, correlation analysis, as well as IHC-based tissue microarrays, also showed that PLEKHA4 had strongly positive correlation with M2 macrophage markers, but weakly with M1 macrophage markers, further solidifying the relationship between PLEKHA4 and M2 macrophage polarization. M2 macrophage infiltration usually predicted a poor outcome in LGG patients. For instance, Liu et al. found that DNTTIP2 expression was associated with M2 macrophage activation and angiogenesis, which in turn causing an unfavorable prognosis in LGG (65). Zhang et al. reported that TP53 R273C mutation promoted the polarization of TAM toward M2 macrophage, leading to an immunosuppressive TME and poor prognosis in LGG (66). In the current study, M2 macrophage infiltration was also found to be negatively correlated with the cumulative survival in LGG, indicating that PLEKHA4 may affect the prognosis of LGG by regulating the polarization and infiltration of M2 macrophages.

Another important finding in this study is that PLEKHA4 modifies the compositions of stromal cells and soluble factors in the TME of LGG patients. In particular, PLEKHA4 expression is significantly correlated with the infiltration of cancer associated fibroblast, which can secrete chemokines and cytokines that promote tumorigenesis (67). What is more, PLEKHA4 expression is also positively correlated to the expression of chemokines (CCL5, CXCL16, CXCL10, CXCL9, CXCL11, etc.), interleukins (IL16, IL10, IL27, IL6, etc.), interferons (IFNG, etc.), and their corresponding receptors (CCR5, CCR1, CXCR3, CXCR6, IFNGR2 etc.). Chemokines, secreted by a variety of cells including TAMs, neutrophils, cancer associated fibroblasts, tumor cells, etc., support many tumor-sustaining processes such as tumor growth, angiogenesis and metastasis (68). Especially, CCR5/CCL5 and CXCR6/CXCL16 signaling modulates TAM polarization, as well as the proliferation and invasion of glioma cells (69, 70). CXCR3/CXCL9/CXCL10/CXCL11 axis leads to recruitment of tumor-promoting immune cells, including TAMs, T cells, etc., thus favoring tumor growth and metastasis (69, 71). Moreover, interleukins can also nurture an environment enabling cancer growth (72). To be specific, IL6 can activate carcinogenesis and tumor outgrowth, and mediate cytokine release syndrome (73, 74). IL10 promotes cytotoxicity but inhibits antitumor responses (75). IFNGR2, the receptor for interferon-γ, was recently identified as a prognostic-related biomarkers correlated with immune infiltration in LGG (76). The release of excessive interleukins, chemokines, and interferons coordinates immune responses, leading to unfavorable tumor behaviors and prognosis. Likewise, enrichment analysis based on DEGs also revealed that PLEKHA4 was potentially involved in the cytokine-cytokine receptor interaction, interferon gamma response, etc., further supported the aforementioned findings. Taken together, PLEKHA4 might play a pivotal role in reshaping the TME in LGG patients, leading to profoundly immunosuppression.

The dynamics of the TME is a superior predictor for therapy response, and knowing the TME can help optimizing immunotherapy (77). In recent years, ICBs therapy has made a breakthrough in the field of tumor immunotherapy (1, 78). However, only a small proportion of glioma patients can respond to ICBs therapy, and the main reason might be the limitations in their tumor immunity status (1, 78). To determine whether PLEKHA4 was capable of predicting the efficiency of ICBs therapy in LGG patients, correlation analysis, as well as TIDE analysis, was performed. We found that PLEKHA4 expression was closely correlated with the expression of immune checkpoints CTLA4, PD-L1, PDCD1, HAVCR2, PDCD1LG2, etc. Meanwhile, the high-PLEKHA4 group achieved higher TIDE scores, suggesting that LGG patients with higher PLEKHA4 expression might have an impaired response to anti-PD-1 or anti-CTLA4 immunotherapy, and PLEKHA4 could act as a predictive biomarker for the ICBs therapy in LGG. It was reported that M2 macrophages are capable of releasing various inhibitory cytokines (IL10, TGF-β, etc.) which further impair the antitumor function of T cells (79, 80). M2 macrophage infiltration might be main reason that causing T cell exclusion and dysfunction.

Additionally, TME features have also been verified to affect drug sensitivity (81). We found that PLEKHA4 expression was positively correlated with the IC50s of B-Raf, ERK and MEK inhibitors (PLX-4720, Vemurafenib, etc.), but negatively with Pyrazoloacridine, SAR-20347, and Palbociclib, etc. Pyrazoloacridine was reported to be a novel dual inhibitor of human topoisomerase I and II with broad antitumor activity (82). SAR-20347, an inhibitor of JAK1, JAK2, JAK3 and TYK2, has been developed as anti-cytokine therapy (83). Palbociclib, an oral inhibitor of CDK4/6, has also been pre-clinically identified as an effective option for glioma (84). Despite the limitations inherent to a small and heterogeneous cohort, this experience suggests that Pyrazoloacridine, SAR-20347 and Palbociclib represent some treatment options for LGG patients with a high level of PLEKHA4.

There were still some limitations in our study. Firstly, the data analyzed in our study were obtained mostly from publicly available datasets. The predicted results of immunotherapy were not verified due to the lack of appropriate glioma immunotherapy cohorts. Furthermore, the conclusions obtained from the limited bioinformatics analysis are insufficient, and further comprehensive experiments and clinical studies should be carried out to verify whether PLEKHA4 exerts such functions.

In conclusion, although some researchers indicated that PLEKHA4 was a prognostic biomarker that was closely correlated with immune infiltration in LGG patients (32, 33), the mechanism underlying PLEKHA4 dysregulation was still not well understood. Meanwhile, the prognostic value and biological role of PLEKHA4 in reshaping TME have not been rigorously studied. Through comprehensive and robust computational studies, we found that PLEKHA4 expression and clinical prognosis of LGG patients are closely related to the CpG methylation status. The synthetical analysis of CpG methylation and PLEKHA4 expression achieves better performance than any single index in predicting OS of LGG patients. Meanwhile, multidimensional bioinformatics analysis, IHC-based tissue microarrays and M2 macrophages infiltration assay *in vitro* revealed that PLEKHA4 reshapes the immunosuppressive TME mainly by affecting the polarization and infiltration of M2 macrophages. Moreover, PLEKHA4 has been proven to be of great significance in predicting the effiiency of immunotherapy and drug sensitivities, allowing clinicians to identify the best management for each patient. These findings help to elucidate the role of PLEKHA4 in carcinogenesis and lay a foundation for further studies.

## Data availability statement

The datasets presented in this study can be found in online repositories. The names of the repository/repositories and accession number(s) can be found in the article/**Supplementary Material**.

## Ethics statement

The studies involving human participants were reviewed and approved by Ethics Committee of Shanghai Outdo Biotech Company. Written informed consent for participation was not required for this study in accordance with the national legislation and the institutional requirements.

## Author contributions

WZ and YW performed experimental study, the data analysis and visualization. CJ and YG were responsible for the data interpretation. QC undertook the statistical analyses. SZ and XM designed the research, prepared the draft, and provided the fund. WD participated in the design and revised the manuscript. All authors contributed to the article and approved the submitted version.
