# Supplementary material for: PLEKHA4 is a novel prognostic biomarker that reshapes the tumor microenvironment in lower-grade glioma
Source: Front Immunol. 2023 Sep 25;14:1128244. doi: 10.3389/fimmu.2023.1128244 (PMC10560889; doi:10.3389/fimmu.2023.1128244)
Supplement: Supplementary file 1 [file Image_1.pdf]

Supplementary Material

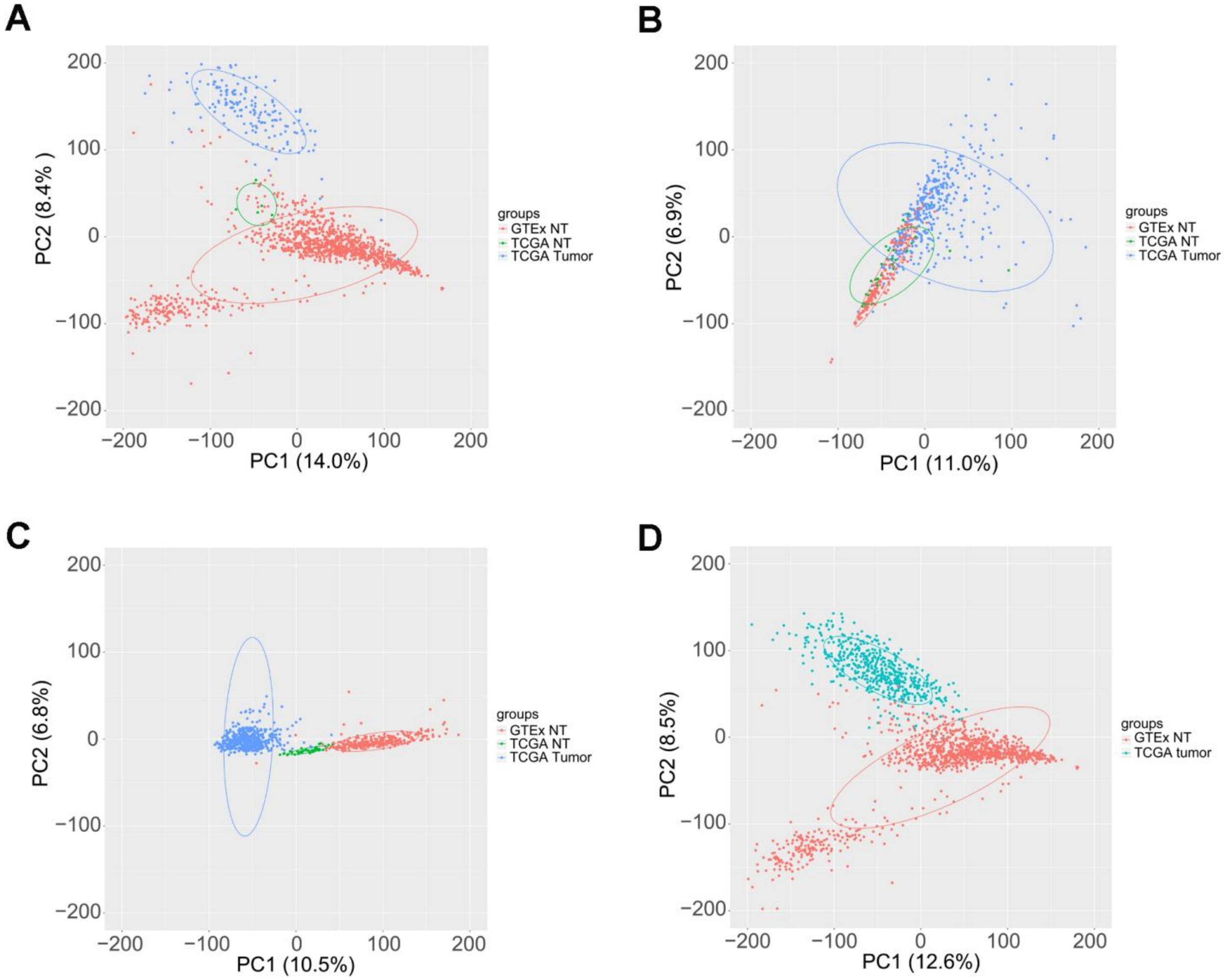

Supplementary Figure 1. PCA plot of cancer and matched normal tissue samples from TCGA and GTEX using RNA-Seq data downloaded from UCSC XENA database, which were processed by the Toil process into TPM format. **(A)** GBM. **(B)** STAD. **(C)** LUSC. **(D)** LGG.

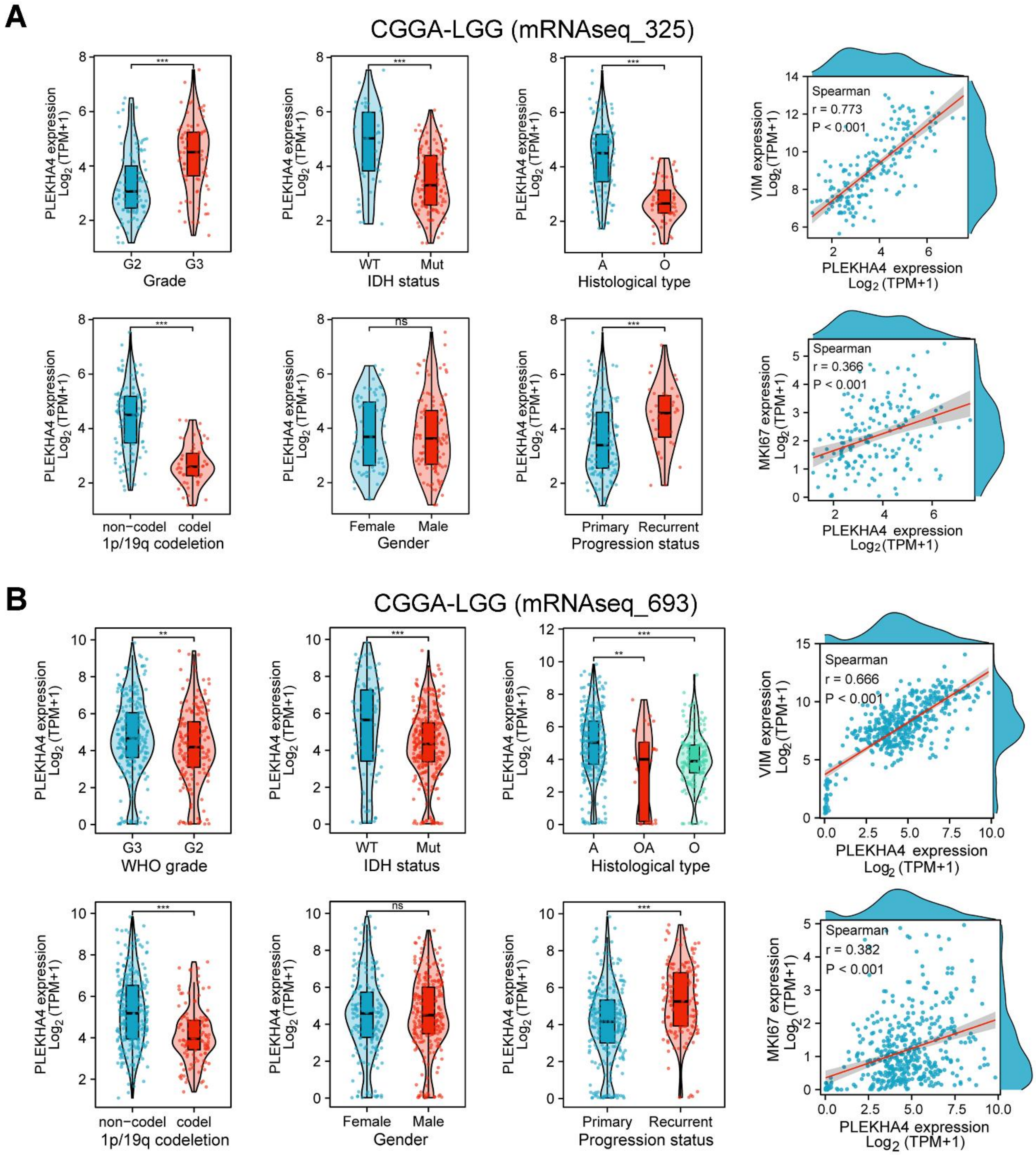

Supplementary Figure 2. Associations between PLEKHA4 expression and different clinical characteristics in **(A)** CGGA-LGG **(**mRNAseq_325**)** cohort and **(B)** CGGA-LGG **(**mRNAseq_693**)** cohort. WT: IDH wild-type; Mut: IDH mutant; A: Astrocytoma; OA: Oligoastrocytomas; O: Oligodendrocytoma; Scatterplots showing the expression correlations of PLEKHA4 and VIM (or MKI67) in the corresponding cohorts. ns, $p \geq 0.05$, $^{**}p < 0.01$, $^{***}p < 0.001$.

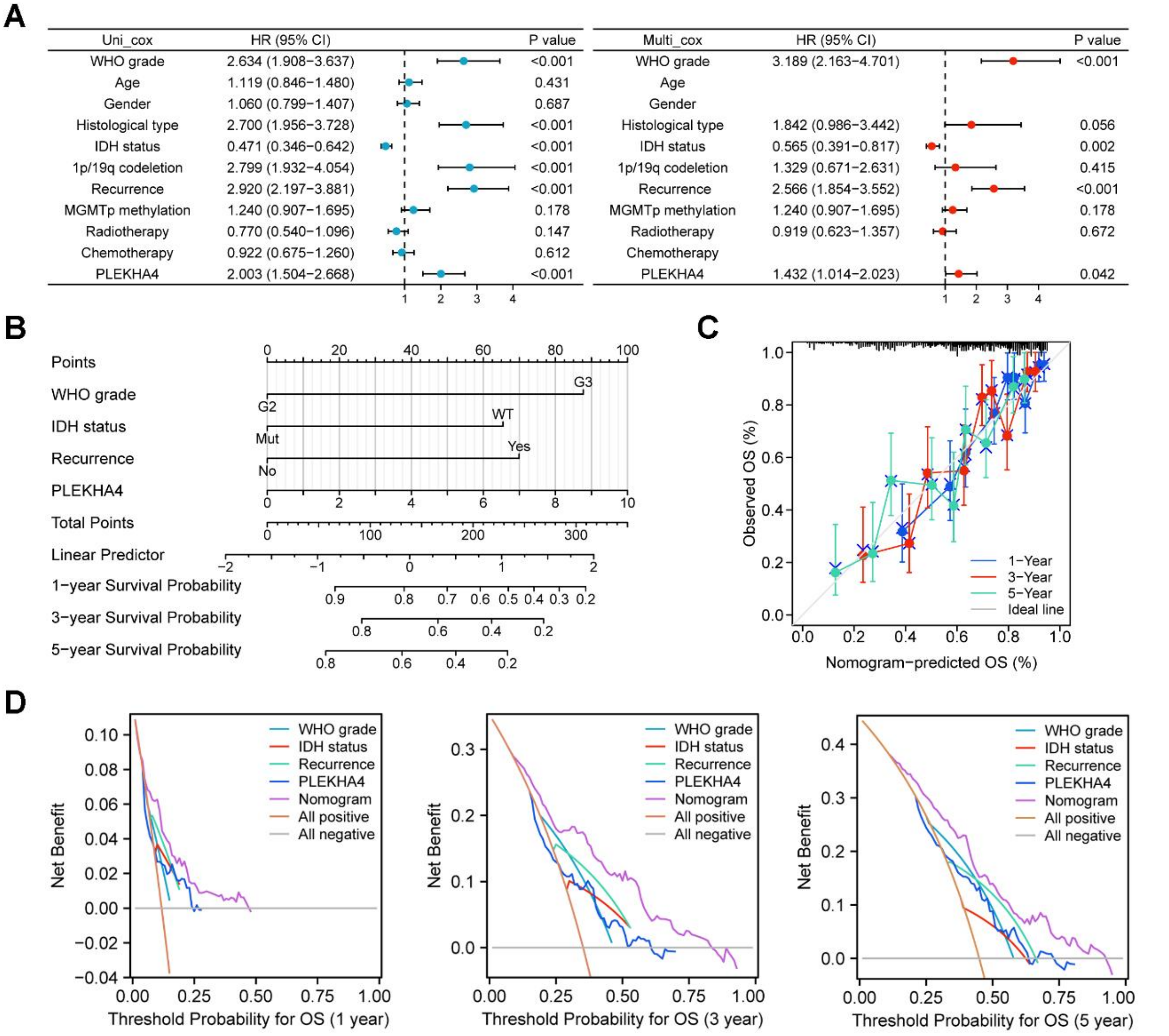

Supplementary Figure 3. Prognostic value of PLEKHA4 in CGGA-LGG (mRNAseq_693) cohort. **(A)** Univariate and multivariate Cox regression analyses of PLEKHA4 expression and other clinical pathological factors for OS of LGG patients. **(B)** Nomogram was constructed with PLEKHA4 expression and clinicopathologic variables to estimate 1-, 3- and 5- OS of LGG patients. **(C)** Calibration plots to verify the accuracy of the predicted 1-, 3- and 5- OS of LGG patients in the nomogram. **(D)** DCA curves to evaluate the accuracy and clinical applicability of the nomogram model for 1-, 3- and 5- OS of LGG patients. WHO grade, IDH status, Recurrence and PLEKHA4 curves represent its own prognostic value. Nomogram curve represents the synthetical prognostic value of the abovementioned factors. All positive curve represents the theoretical best prognostic value, and all negative curve represents no prognostic value.

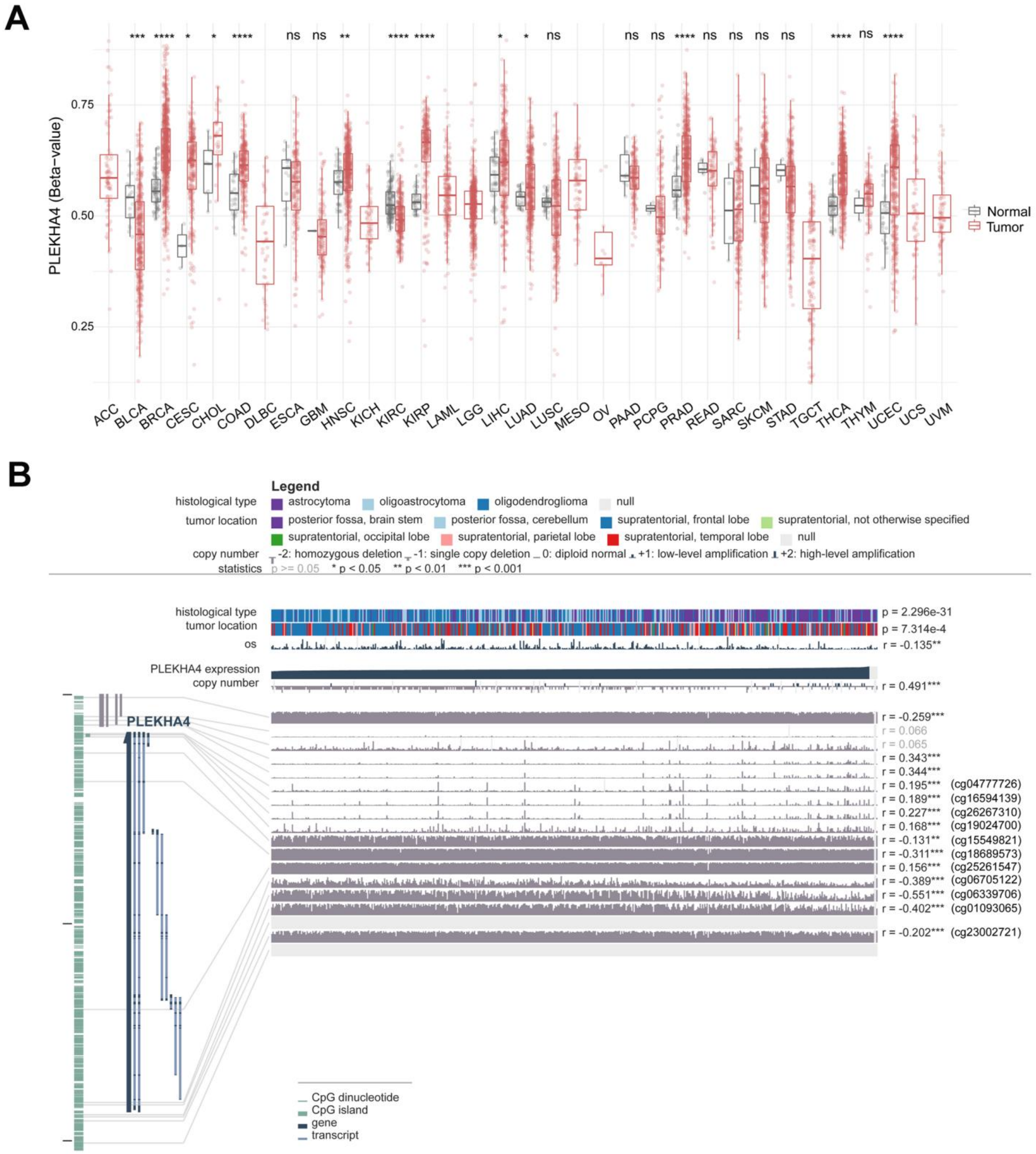

Supplementary Figure 4. (A) Pan-cancer methylation profiles of PLEKHA4 from SMART App. **(B)** Integration and visualization of PLEKHA4 expression and DNA methylation in combination with the precise genomic location of the CpG sites using MEXPRESS.

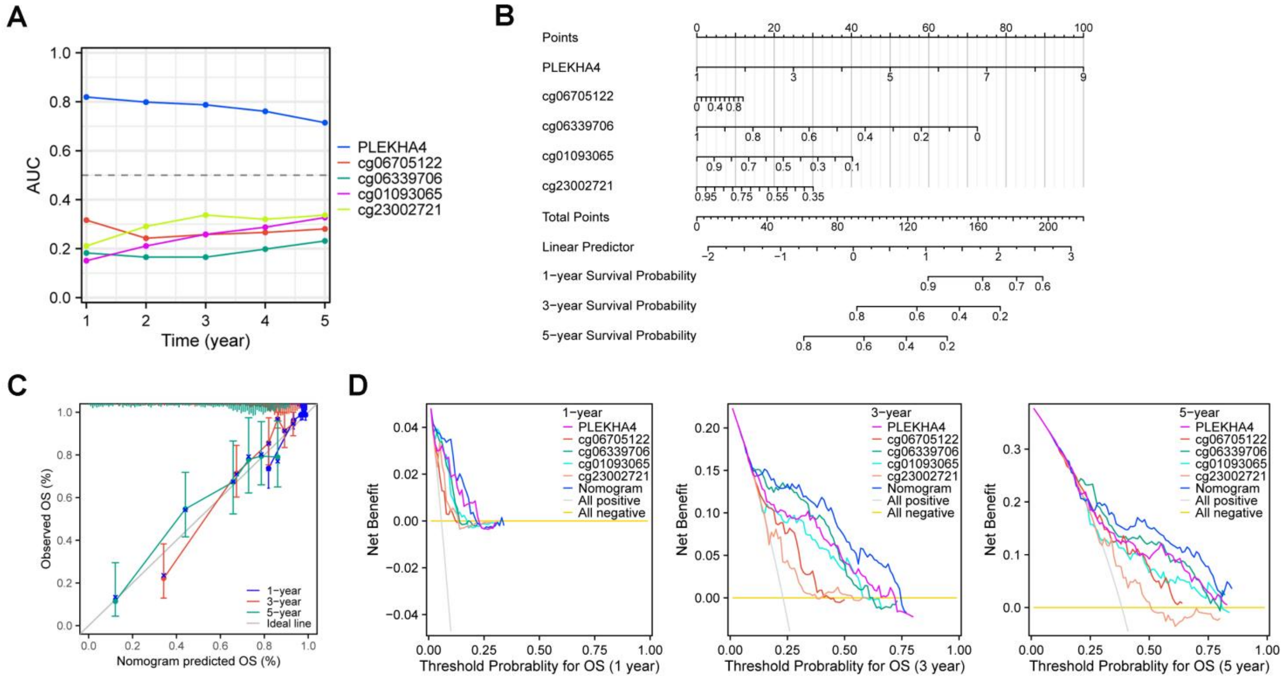

Supplementary Figure 5. The combined analysis of CpG methylation and PLEKHA4 expression is more helpful for predicting the OS of LGG patients. **(A)** AUC curves to illustrate the discriminative ability of PLEKHA4 and four CpG sites in predicting 1-5 OS. **(B)** Nomogram was constructed with PLEKHA4 expression and four CpG sites to estimate 1-, 3- and 5- OS of LGG patients. **(C)** Calibration plots to verify the accuracy of the predicted 1-, 3- and 5- OS of LGG patients in the nomogram. **(D)** DCA curves to evaluate the accuracy and clinical applicability of the nomogram model for 1-, 3- and 5- OS of LGG patients. PLEKHA4, cg06705122, cg06339706, cg01093065 and cg23002721 curves represent its own prognostic value. Nomogram curve represents the synthetical prognostic value of the abovementioned factors. All positive curve represents the theoretical best prognostic value, and all negative curve represents no prognostic value.

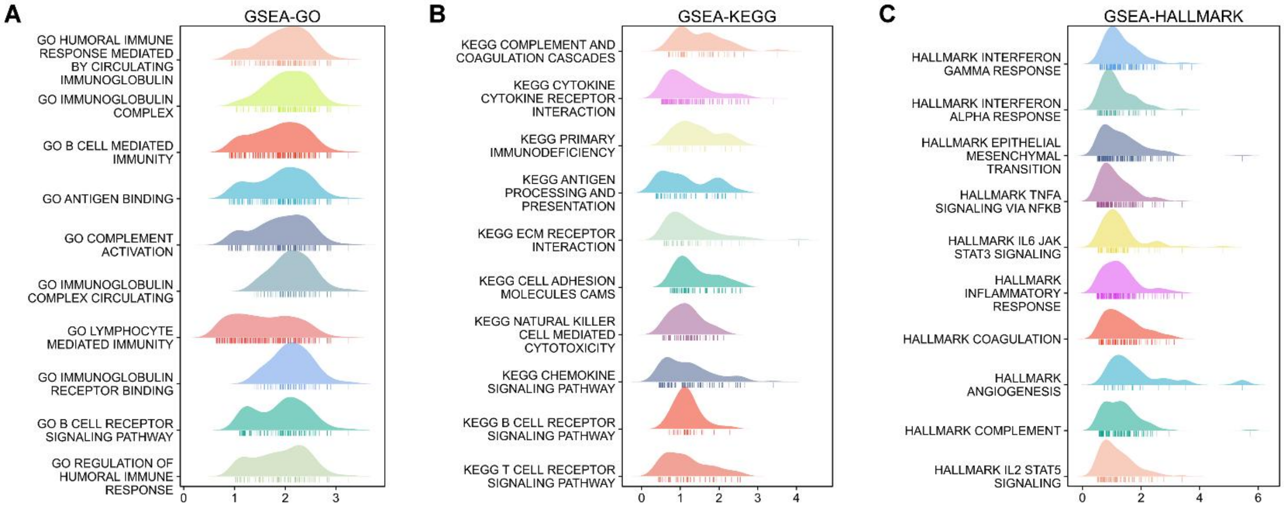

Supplementary Figure 6. Merged plots of GSEA showing the signaling pathways associated with PLEKHA4 1040 expression according to (A) GO, (B) KEGG, and (C) Hallmark analyses in LGG.

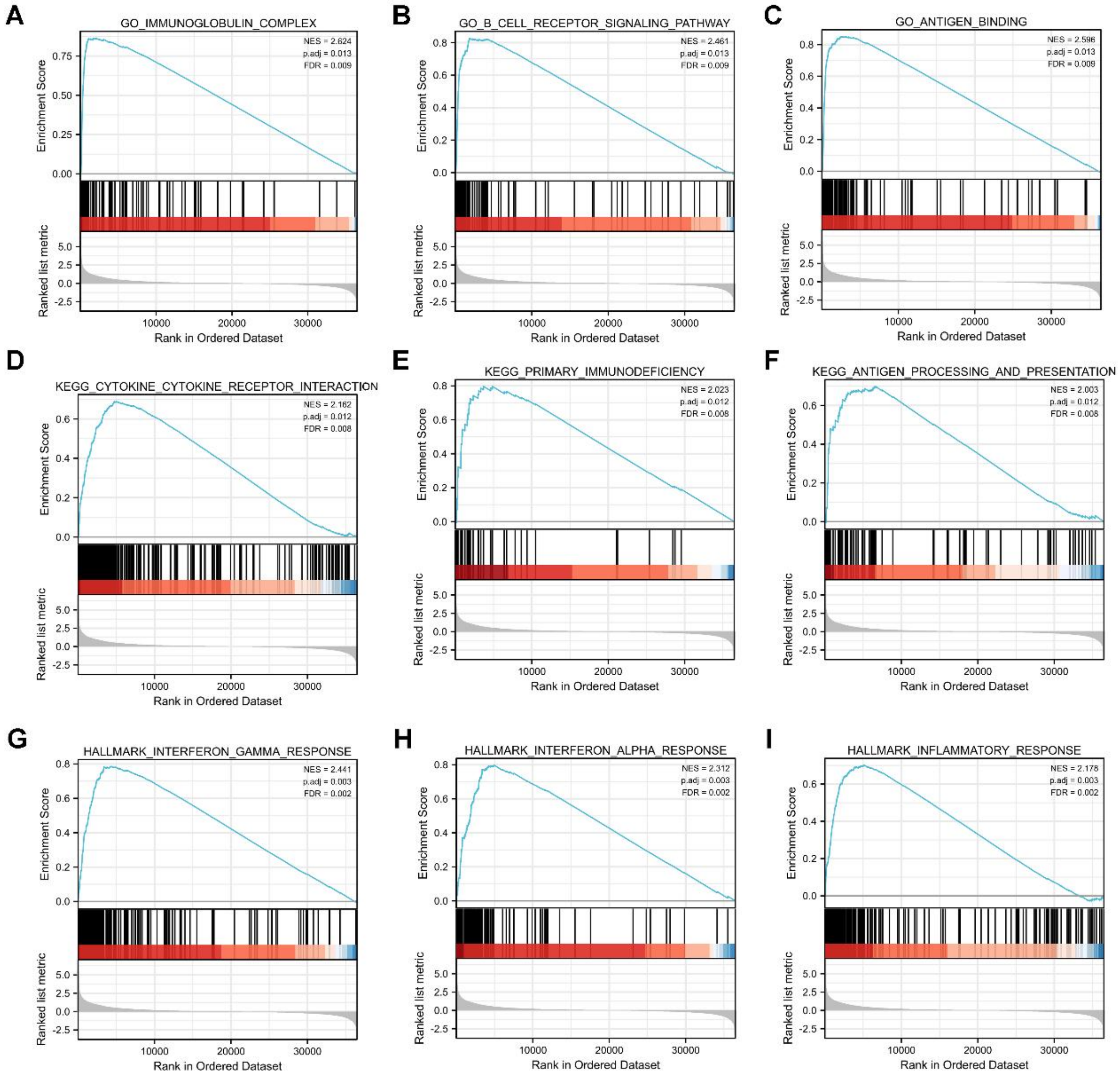

Supplementary Figure 7. Functional enrichment of PLEKHA4 in LGG by GSEA. **(A)** Enrichment of genes in the immunoglobulin complex by GSEA. **(B)** Enrichment of genes in the B cell receptor signaling pathway by GSEA. **(C)** Enrichment of genes in the antigen binding pathway by GSEA. **(D)** Enrichment of genes in the cytokine-cytokine receptor interaction pathway by GSEA. **(E)** Enrichment of genes in the primary immunodeficiency pathway by GSEA. **(F)** Enrichment of genes in the antigen processing and presentation pathway by GSEA. **(G)** Enrichment of genes in the interferon gamma response by GSEA. **(H)** Enrichment of genes in the interferon alpha response by GSEA. **(I)** Enrichment of genes in the inflammatory response by GSEA.

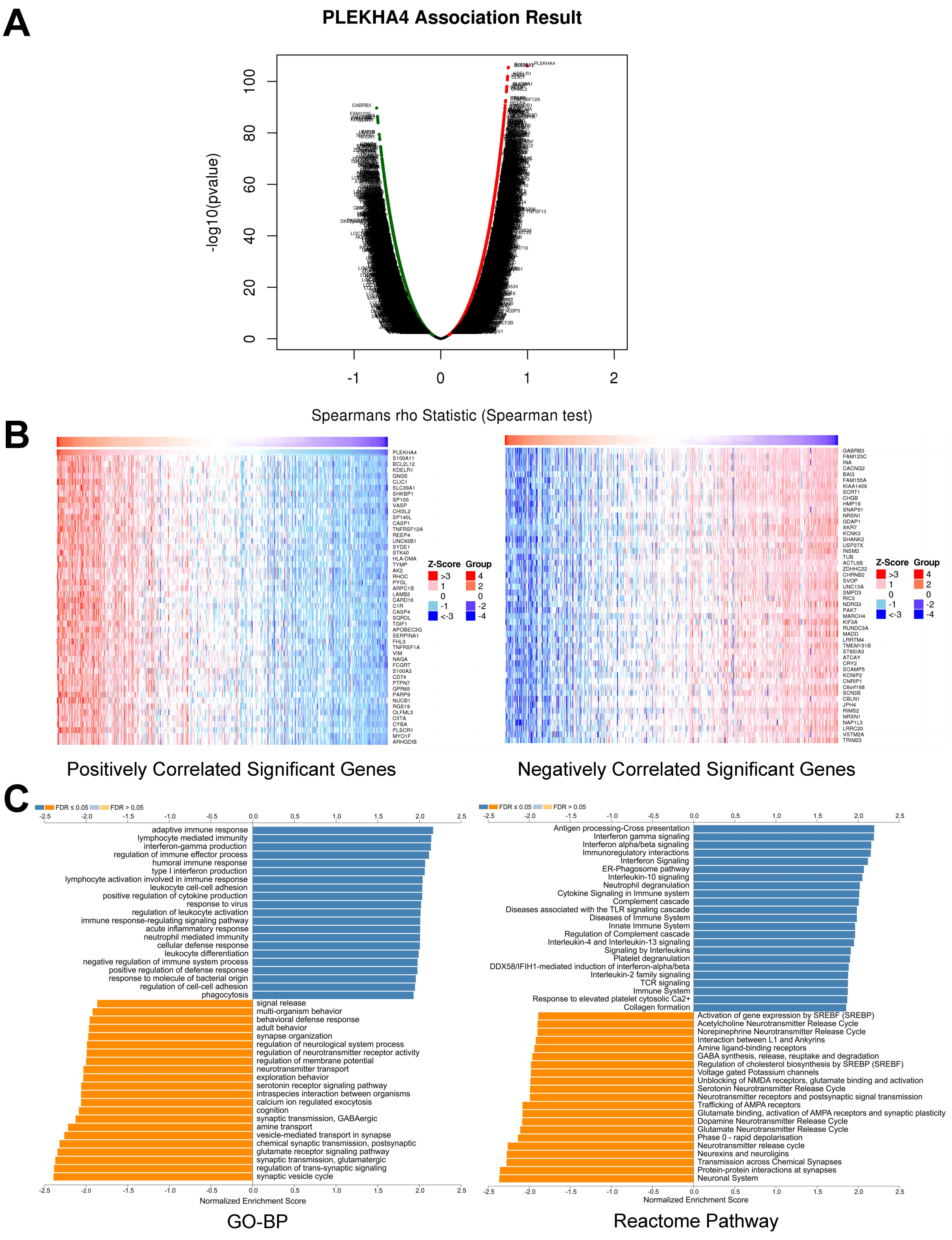

Supplementary Figure 8. The coexpression network of PLEKHA4 in LGG. **(A)** The volcano plot of coexpressed genes. **(B)** Heatmaps of top 50 genes positively and negatively related to PLEKHA4. **(C)** GO terms for biological process and reactome pathways of PLEKHA4-coexpressed genes by GSEA analyses.

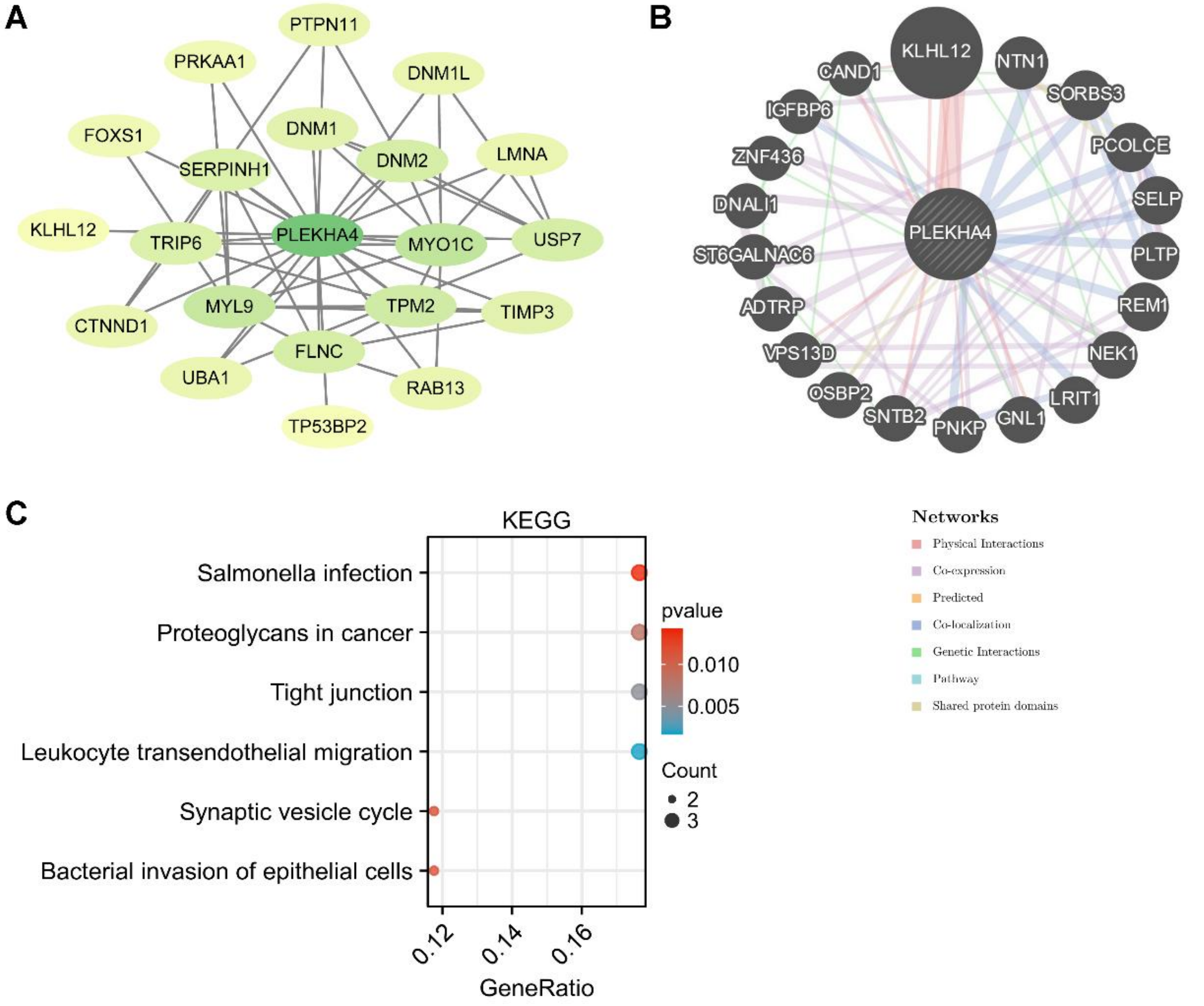

Supplementary Figure 9. Interaction network of PLEKHA4 in LGG. **(A)** The PPI network of PLEKHA4 constructed by STRING. **(B)** PLEKHA4 related gene-gene interaction network obtained from GeneMANIA. **(C)** KEGG enrichment analysis of PLEKHA4-binding partners.

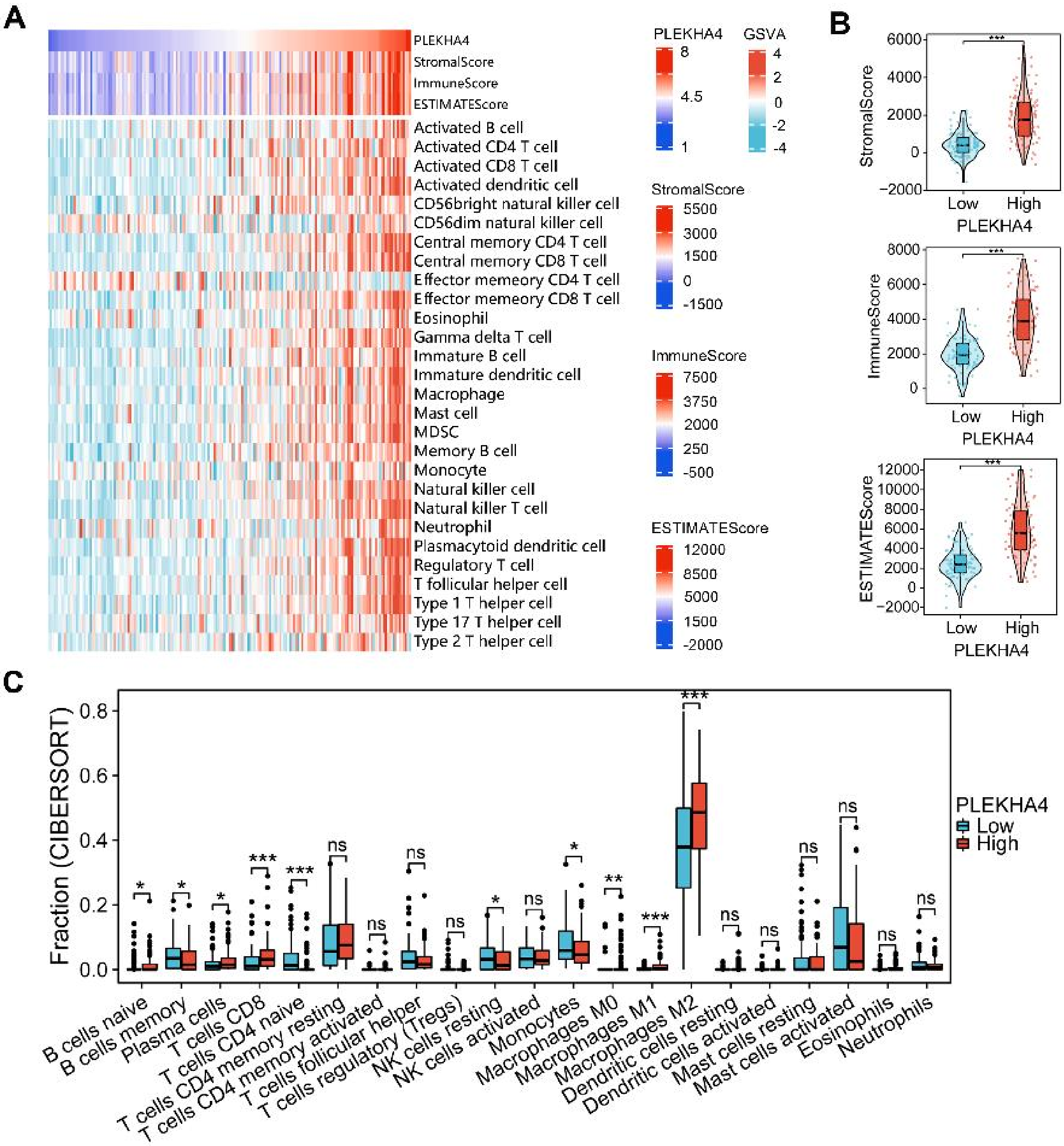

Supplementary Figure 10. PLEKHA4 correlated with tumor immunity in CGGA-LGG (mRNAseq_325) cohort. **(A)** Heatmaps showing the association between PLEKHA4 expression and tumor purity (ESTIMATE algorithm), as well as the tumor-infiltrating immune cells (ssGSEA algorithm), in the CGGA-LGG (mRNAseq_325) cohort. **(B)** The comparison of StromalScore, ImmuneScore, and ESTIMATEScore between the high- and low- PLEKHA4 groups in the CGGA-LGG (mRNAseq_325) cohort. **(C)** Comparison of tumor-infiltrating immune cells between the high- and low- PLEKHA4 groups. ns, $p \geq 0.05$, *$p < 0.05$, **$p < 0.01$, ***$p < 0.001$.

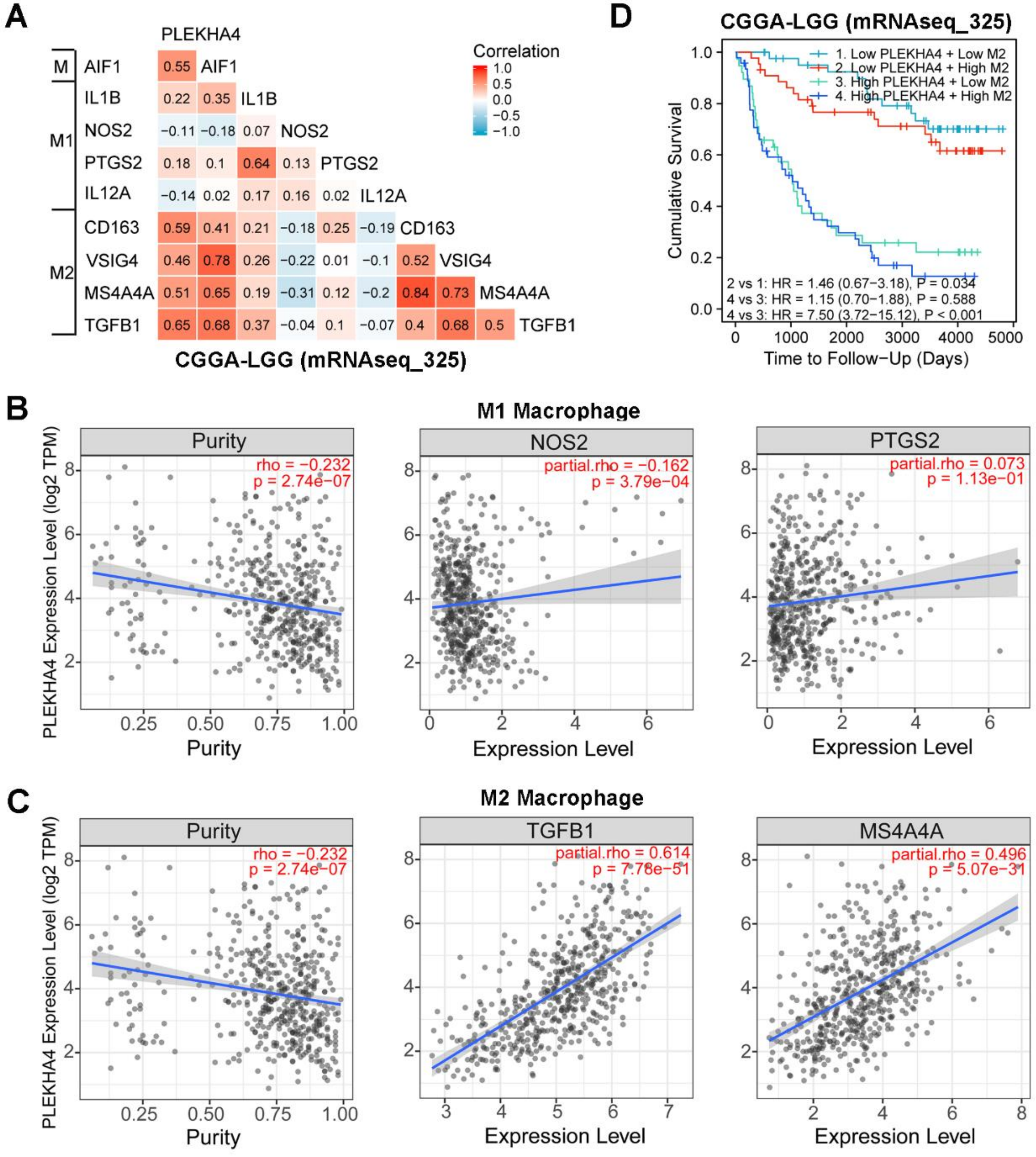

Supplementary Figure 11. The role of PLEKHA4 in regulating the polarization and infiltration of M2 macrophages, based on CGGA-LGG (mRNAseq_325) cohort. **(A)** The heat maps showing the correlations of PLEKHA4 and phenotype markers of monocytes (AIF1), M1 macrophages (IL1B, NOS2, PTGS2, IL12A), and M2 macrophages (CD163, VSIG4, TGFB1, MS4A4A). **(B)** Associations of the PLEKHA4 expression with M1 macrophage markers NOS2 and PTGS2 obtained from the TIMER database. **(C)** Associations of the PLEKHA4 expression with M2 macrophage markers TGFB1 and MS4A4A by the TIMER database. **(D)** Kaplan‑Meier survival analysis

showing the prognostic significance of PLEKHA4 based on M2 macrophage infiltration in the CGGA-LGG (mRNAseq_325) cohort.

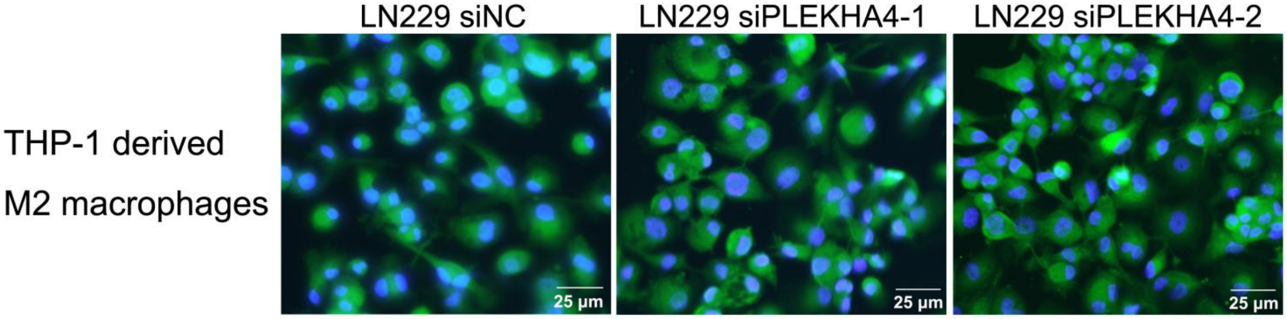

Supplementary Figure 12. Identification of THP-1 derived M2 macrophages using immunofluorescence staining. More than 98% cells were strongly positive for CD163, indcating that the THP-1 cells have been successfully polarized into M2 macrophages.

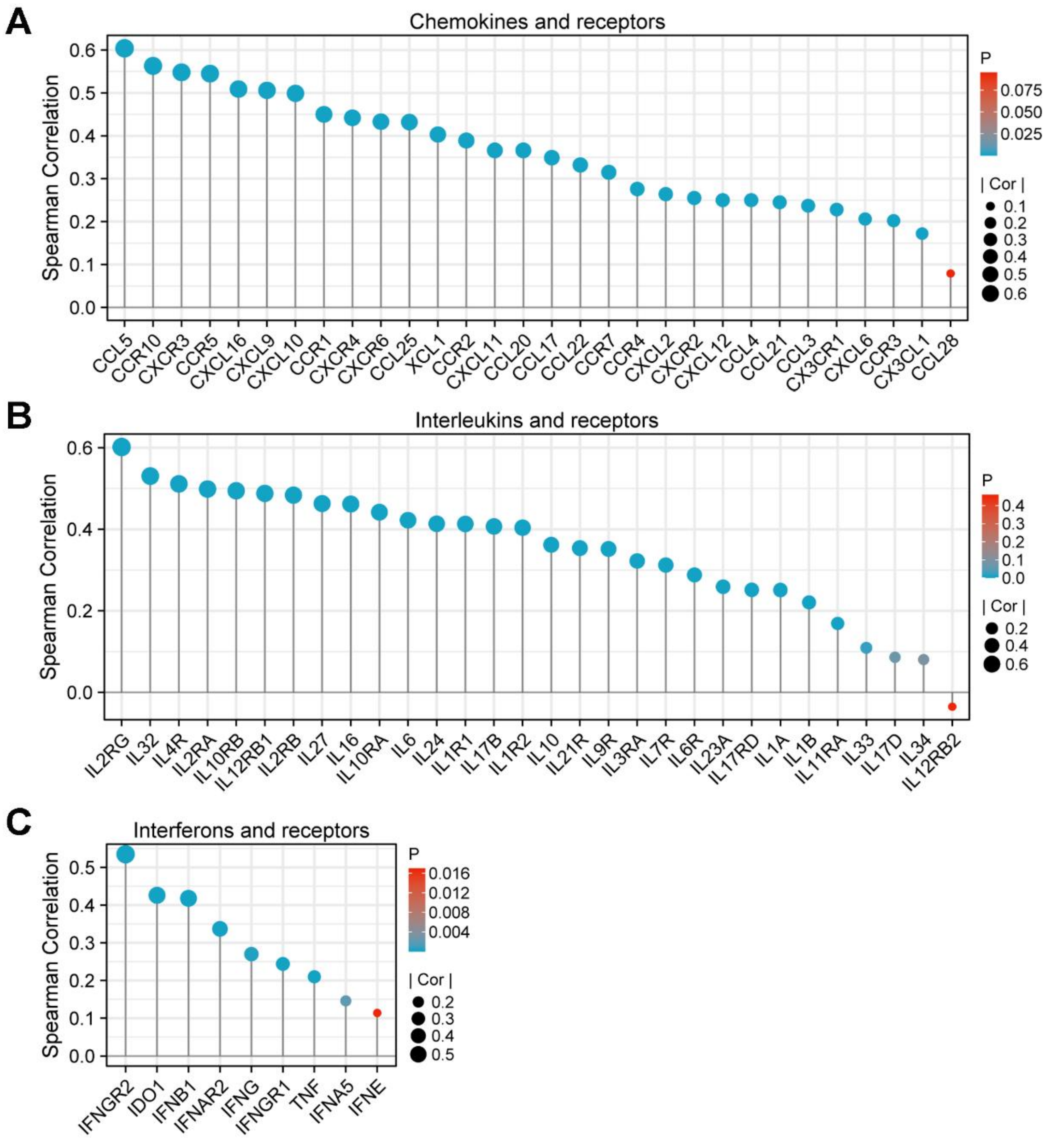

Supplementary Figure 13. Association between PLEKHA4 expression and microenvironment of LGG. **(A-C)** Correlation anasysis between PLEKHA4 and **(A)** cytokines, **(B)** interleukins or **(C)** interferons, as well as their corresponding receptors in the CGGA-LGG (mRNAseq_693) cohort.

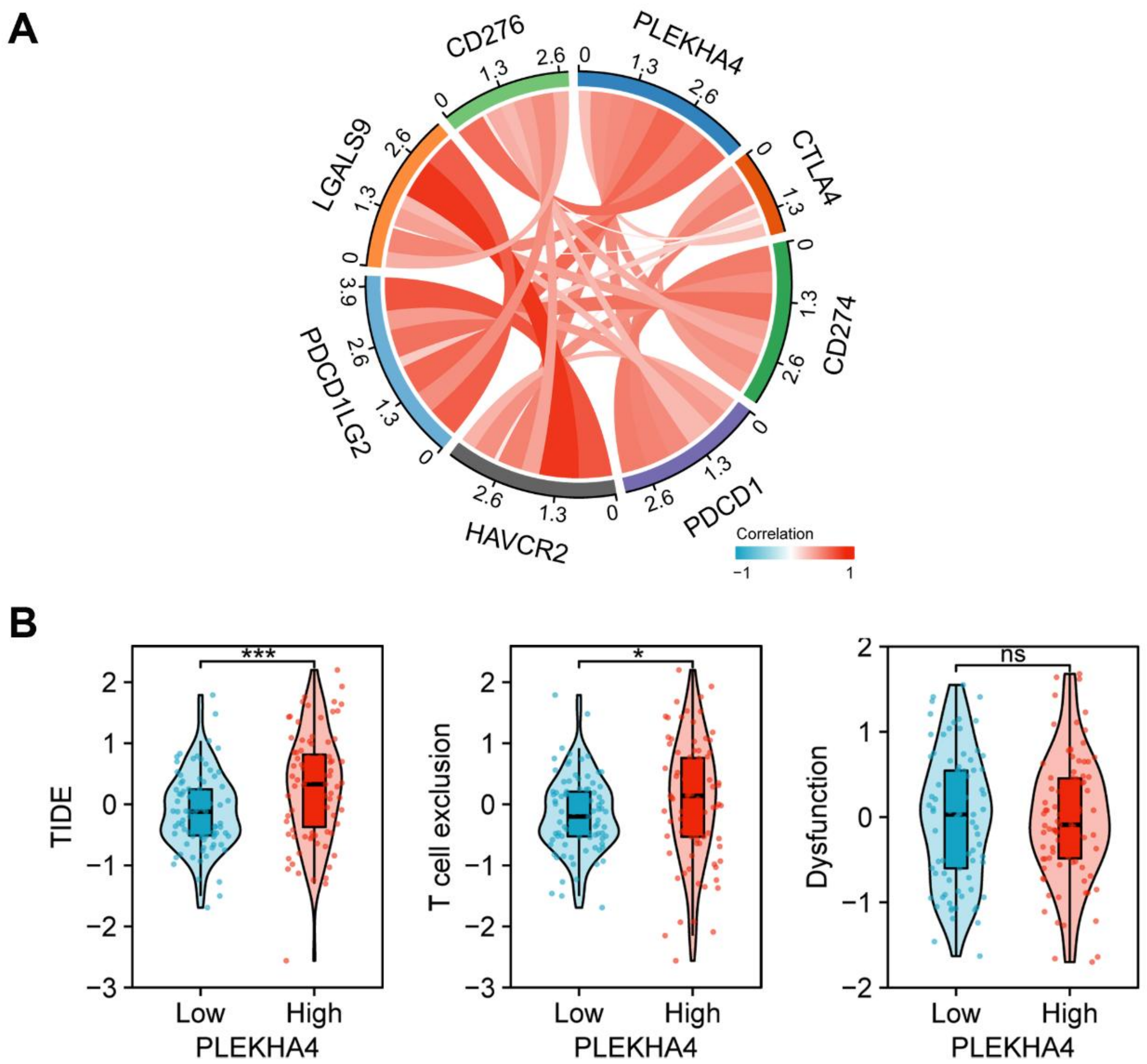

Supplementary Figure 14. Clinical significance of PLEKHA4 in guiding immunotherapy, based on CGGA-LGG (mRNAseq_325) cohort. **(A)** Chord diagram showing the correlations of PLEKHA4 and immune-related checkpoints. **(B)** Comparison of TIDE, T cell exclusion and dysfunction scores between the high- and low- PLEKHA4 groups. ns, $p \geq 0.05$, $*p < 0.05$, $***p < 0.001$.
