# Supplementary material for: PLEKHA4 is a novel prognostic biomarker that reshapes the tumor microenvironment in lower-grade glioma
Source: Front Immunol. 2023 Sep 25;14:1128244. doi: 10.3389/fimmu.2023.1128244 (PMC10560889; doi:10.3389/fimmu.2023.1128244)
Supplement: Supplementary file 2 [file Table_1.pdf]

Supplementary Material

Supplementary Table 1. Demographic and clinical characteristics of LGG patients with low and high expression of PLEKHA4 in TCGA (n=528).

**Characteristic**	**levels**	**PLEKHA4 expression**		***p***
		**Low (n=264)**	**High (n=264)**	
Age, n (%)	<=40	137 (25.9%)	127 (24.1%)	0.433
	>40	127 (24.1%)	137 (25.9%)	
Gender, n (%)	Female	124 (23.5%)	115 (21.8%)	0.484
	Male	140 (26.5%)	149 (28.2%)	
WHO grade, n (%)	G2	143 (30.6%)	81 (17.3%)	< 0.001
	G3	94 (20.1%)	149 (31.9%)	
Histological type, n (%)	Astrocytoma	51 (9.7%)	144 (27.3%)	< 0.001
	Oligoastrocytoma	65 (12.3%)	69 (13.1%)	
	Oligodendroglioma	148 (28%)	51 (9.7%)	
IDH status, n (%)	WT	13 (2.5%)	84 (16%)	< 0.001
	Mut	249 (47.4%)	179 (34.1%)	
1p/19q codeletion, n (%)	codel	147 (27.8%)	24 (4.5%)	< 0.001
	non-codel	117 (22.2%)	240 (45.5%)	
Primary therapy outcome, n (%)	PD	38 (8.3%)	72 (15.7%)	0.002
	SD	78 (17%)	68 (14.8%)	
	PR	31 (6.8%)	33 (7.2%)	
	CR	80 (17.5%)	58 (12.7%)	
Laterality, n (%)	Left	126 (24.1%)	130 (24.9%)	0.676
	Midline	2 (0.4%)	4 (0.8%)	
	Right	133 (25.4%)	128 (24.5%)	
Race, n (%)	Asian	5 (1%)	3 (0.6%)	0.369
	Black or African American	8 (1.5%)	14 (2.7%)	
	White	245 (47.4%)	242 (46.8%)	
OS event, n (%)	Alive	223 (42.2%)	169 (32%)	< 0.001
	Dead	41 (7.8%)	95 (18%)	

DSS event, n (%)	Alive	224 (43.1%)	173 (33.3%)	< 0.001
	Dead	37 (7.1%)	86 (16.5%)	
PFI event, n (%)	Alive	184 (34.8%)	134 (25.4%)	< 0.001
	Dead	80 (15.2%)	130 (24.6%)	

Note: Chi‐ square tests were used to evaluate age, gender, WHO grade, histological type, IDH status, 1p/19q codeletion, primary therapy outcome, OS event, DSS event and PFI event. Fisher's exact tests were used to evaluate laterality and race.

Supplementary Table 2. Demographic and clinical characteristics of LGG patients with low and high expression of PLEKHA4 in CGGA (mRNAseq_693, n(LGG)=443).

Characteristic	levels	PLEKHA4 expression		*p*
		Low (n=221)	High (n=222)	
Age, n (%)	<=40	116 (26.2%)	112 (25.3%)	0.775
	>40	105 (23.8%)	109 (24.7%)	
Gender, n (%)	Female	96 (21.7%)	96 (21.7%)	1.000
	Male	125 (28.2%)	126 (28.4%)	
WHO grade, n (%)	G2	108 (24.4%)	80 (18.1%)	0.008
	G3	113 (25.5%)	142 (32.1%)	
Histological type, n (%)	Astrocytoma	115 (26%)	156 (35.2%)	< 0.001
	Oligoastrocytoma	14 (3.2%)	16 (3.6%)	
	Oligodendroglioma	92 (20.8%)	50 (11.3%)	
IDH status, n (%)	WT	37 (9.2%)	59 (14.7%)	0.003
	Mut	173 (43%)	133 (33.1%)	
1p/19q codeletion, n (%)	codel	80 (19.8%)	51 (12.6%)	< 0.001
	non-codel	104 (25.7%)	169 (41.8%)	
Recurrence, n (%)	NO	162 (36.6%)	120 (27.1%)	< 0.001
	Yes	59 (13.3%)	102 (23%)	
MGMTp methylation, n (%)	Yes	138 (40.2%)	71 (20.7%)	0.226
	NO	79 (23%)	55 (16%)	
Radiotherapy, n (%)	NO	55 (13.2%)	47 (11.3%)	0.510
	Yes	156 (37.4%)	159 (38.1%)	
Chemotherapy, n (%)	NO	57 (13.7%)	75 (18%)	0.068
	Yes	152 (36.5%)	133 (31.9%)	

Note: Chi- square tests were performed to clarify the differences in clinicopathological characteristics among samples.

Supplementary Table 3. Demographic and clinical characteristics of LGG patients with low and high expression of PLEKHA4 in CGGA (mRNAseq_325, n(LGG)=182).

**Characteristic**	**levels**	**PLEKHA4 expression**		***p***
		**Low (n=91)**	**High (n=91)**	
Age, n (%)	<=40	53 (29.1%)	49 (26.9%)	0.654
	>40	38 (20.9%)	42 (23.1%)	
Gender, n (%)	Female	36 (19.8%)	35 (19.2%)	1.000
	Male	55 (30.2%)	56 (30.8%)	
WHO grade, n (%)	G2	69 (37.9%)	34 (18.7%)	< 0.001
	G3	22 (12.1%)	57 (31.3%)	
Histological type, n (%)	Astrocytoma	27 (14.8%)	29 (15.9%)	< 0.001
	Anaplastic astrocytoma	10 (5.5%)	52 (28.6%)	
	Anaplastic oligodendroglioma	9 (4.9%)	3 (1.6%)	
	Oligodendroglioma	45 (24.7%)	7 (3.8%)	
IDH status, n (%)	Mut	79 (43.6%)	54 (29.8%)	< 0.001
	WT	11 (6.1%)	37 (20.4%)	
1p/19q codeletion, n (%)	codel	52 (28.9%)	8 (4.4%)	< 0.001
	non-codel	37 (20.6%)	83 (46.1%)	
Recurrence, n (%)	NO	82 (45.1%)	62 (34.1%)	< 0.001
	Yes	9 (4.9%)	29 (15.9%)	
MGMTp methylation, n (%)	Yes	49 (29.5%)	40 (24.1%)	0.362
	NO	36 (21.7%)	41 (24.7%)	
Radiotherapy, n (%)	NO	15 (8.6%)	17 (9.8%)	0.734
	Yes	74 (42.5%)	68 (39.1%)	
Chemotherapy, n (%)	NO	41 (24.6%)	35 (21%)	0.480
	Yes	43 (25.7%)	48 (28.7%)	

Note: Chi- square tests were performed to clarify the differences in clinicopathological characteristics among samples.

Supplementary Table 4. Demographic and clinical characteristics of GBM patients with low and high expression of PLEKHA4 in TCGA (n=168).

**Characteristic**	**levels**	**PLEKHA4 expression**		***p***
		**Low (n=84)**	**High (n=84)**	
Age, n (%)	<=60	43 (25.6%)	44 (26.2%)	1.000
	>60	41 (24.4%)	40 (23.8%)	
Gender, n (%)	Female	26 (15.5%)	33 (19.6%)	0.332
	Male	58 (34.5%)	51 (30.4%)	
IDH status, n (%)	WT	70 (43.5%)	79 (49.1%)	0.034
	Mut	10 (6.2%)	2 (1.2%)	
Race, n (%)	Asian	2 (1.2%)	3 (1.8%)	0.706
	Black or African American	7 (4.2%)	4 (2.4%)	
	White	75 (45.2%)	75 (45.2%)	
OS event, n (%)	Alive	14 (8.3%)	18 (10.7%)	0.556
	Dead	70 (41.7%)	66 (39.3%)	
DSS event, n (%)	Alive	16 (10.3%)	18 (11.6%)	0.748
	Dead	63 (40.6%)	58 (37.4%)	
PFI event, n (%)	Alive	16 (9.5%)	16 (9.5%)	1.000
	Dead	68 (40.5%)	68 (40.5%)	

Note: Chi- square tests were used to evaluate age, gender, IDH status, OS event, DSS event and PFI event. Fisher's exact test was used to evaluate race.

Supplementary Table 5. Demographic and clinical characteristics of GBM patients with low and high expression of PLEKHA4 in CGGA (mRNAseq_693, n(GBM)=249).

**Characteristic**	**levels**	**PLEKHA4 expression**		***p***
		**Low (n=124)**	**High (n=125)**	
Age, n (%)	<=60	96 (38.6%)	98 (39.4%)	0.973
	>60	28 (11.2%)	27 (10.8%)	
Gender, n (%)	Female	50 (20.1%)	52 (20.9%)	0.847
	Male	74 (29.7%)	73 (29.3%)	
Histological type, n (%)	GBM	76 (30.5%)	64 (25.7%)	0.140
	rGBM	48 (19.3%)	61 (24.5%)	
IDH status, n (%)	Mut	31 (13%)	18 (7.5%)	0.037
	WT	86 (36%)	104 (43.5%)	
1p/19q codeletion, n (%)	codel	9 (4.1%)	4 (1.8%)	0.095
	non-codel	85 (39%)	120 (55%)	
Recurrence, n (%)	NO	76 (30.5%)	64 (25.7%)	0.140
	Yes	48 (19.3%)	61 (24.5%)	
MGMTp methylation, n (%)	Yes	53 (26.6%)	53 (26.6%)	0.813
	NO	44 (22.1%)	49 (24.6%)	
Radiotherapy, n (%)	NO	16 (7%)	18 (7.9%)	0.874
	Yes	98 (42.8%)	97 (42.4%)	
Chemotherapy, n (%)	NO	15 (6.5%)	14 (6.1%)	0.920
	Yes	98 (42.6%)	103 (44.8%)	

Note: Chi- square tests were performed to clarify the differences in clinicopathological characteristics among samples.

Supplementary Table 6. Demographic and clinical characteristics of GBM patients with low and high expression of PLEKHA4 in CGGA (mRNAseq_325, n(GBM)=139).

Characteristic	levels	PLEKHA4 expression		*p*
		**Low (n=70)**	**High (n=69)**	
Age, n (%)	<=60	62 (44.6%)	63 (45.3%)	0.800
	>60	8 (5.8%)	6 (4.3%)	
Gender, n (%)	Female	25 (18%)	26 (18.7%)	0.949
	Male	45 (32.4%)	43 (30.9%)	
Histological type, n (%)	GBM	49 (35.3%)	36 (25.9%)	0.092
	rGBM	10 (7.2%)	14 (10.1%)	
	sGBM	11 (7.9%)	19 (13.7%)	
IDH status, n (%)	Mut	10 (7.2%)	31 (22.3%)	< 0.001
	WT	60 (43.2%)	38 (27.3%)	
1p/19q codeletion, n (%)	codel	1 (0.7%)	6 (4.4%)	0.057
	non-codel	69 (50.7%)	60 (44.1%)	
Recurrence, n (%)	NO	49 (35.3%)	36 (25.9%)	0.047
	Yes	21 (15.1%)	33 (23.7%)	
MGMTp methylation, n (%)	Yes	27 (19.9%)	39 (28.7%)	0.040
	NO	42 (30.9%)	28 (20.6%)	
Radiotherapy, n (%)	NO	18 (13.5%)	15 (11.3%)	0.801
	Yes	50 (37.6%)	50 (37.6%)	
Chemotherapy, n (%)	NO	17 (12.7%)	17 (12.7%)	1.000
	Yes	51 (38.1%)	49 (36.6%)	

Note: Chi- square tests were performed to clarify the differences in clinicopathological characteristics among samples.

Supplementary Material

Supplementary Table 7. Details of the cancer and matched normal tissue samples from TCGA and GTEX studies respectively, downloaded from UCSC Xena.

TCGA cancer abbreviation	TCGA cancer	Primary site of the tumor	Number of normal solid tissues from TCGA	Number of tumor samples From TCGA	Primary site of GTEX samples	GTEX -site with sublocations	Number of healthy normal samples from GTEx
ACC	Adrenocortical carcinoma	Adrenal Gland	0	77	Adrenal Gland	Adrenal Gland	128
BLCA	Bladder Urothelial Carcinoma	Bladder	19	405	Bladder	Bladder	9
BRCA	Breast Invasive Carcinoma	Breast	113	1099	Breast	Breast - Mammary Tissue	179
CESC	Cervical & Endocervical Cancer	Cervix	3	306	Cervix Uteri	Cervix - Ectocervix, Endocervix	10
CHOL	Cholangio carcinoma	Cholangio	9	36	-	-	-
COAD	Colon Adenocarcinoma	Colon	41	290	Colon	Colon - Sigmoid, Transverse	308
DLBC	Lymphoid Neoplasm	Blood	0	47	Blood	Blood	444

	Diffuse Large B-cell Lymphoma						
ESCA	Esophageal Carcinoma	Esophagus	13	182	Esophagus	Esophagus - Gastroesophageal Junction, Mucosa, Muscularis	653
GBM	Glioblastoma Multiforme	Brain	5	166	Brain	Brain - Amygdala, Anterior cingulate cortex (BA24), Caudate(Basal Ganglia), Cerebellar Hemisphere, Cerebellum, Cortex, Frontal Cortex BA9), Hippo campus, Hypothalamus, Nucleus accumbens (basal ganglia), Putamen (basal ganglia), Spinal cord (Cervical c-1), Substanti a Nigra	1152

HNSC	Head and Neck squamous cell carcinoma	Head and Neck squamous cell	44	497	-	-	-
KICH	Kidney Chromophobe	Kidney	25	66	Kidney	Kidney - Cortex	28
KIRC	Kidney Clear Cell Carcinoma	Kidney	72	531	Kidney	Kidney - Cortex	28
KIRP	Kidney Papillary	Kidney	32	289	Kidney	Kidney - Cell Carcinoma Cortex	28
LAML	Acute Myeloid Leukemia	Bone Marrow	0	173	Bone Marrow	Bone Marrow	70

LGG	Brain Lower Grade Glioma	Brain	0	523	Brain	Brain - Amygdala, Anterior cingulate cortex (BA24), Caudate(Basal Ganglia), Cerebellar Hemisphere, Cerebellum, Cortex, Frontal Cortex BA9), Hippo campus, Hypothalamus, Nucleus accumbens (basal ganglia), Putamen (basal ganglia), Spinal cord (Cervical c-1), Substanti a Nigra	1152
LIHC	Liver Hepatocellular Carcinoma	Liver	50	371	Liver	Liver	110
LUAD	Lung Adenocarcinoma	Lung	59	515	Lung	Lung	288
LUSC	Lung Squamous Cell Carcinoma	Lung	50	498	Lung	Lung	288

MESO	Mesothelioma	Mesenchymal	0	87	-	-	-
OV	Ovarian serous cystadenocarcinoma	Ovary	0	427	Ovary	Ovary	88
PAAD	Pancreatic Adenocarcinoma	Pancreas	4	179	Pancreas	Pancreas	167
PCPG	Pheochromocytoma and Paraganglioma	Adrenal Gland	3	182	-	-	-
PRAD	Prostate Adenocarcinoma	Prostate	52	496	Prostate	Prostate	100
READ	Rectum Adenocarcinoma	Rectum	10	93	Colon	Colon - Sigmoid, Transverse	308
SARC	Sarcoma	Mesenchymal	2	262	-	-	-
SKCM	Skin Cutaneous Melanoma	Skin	1	469	Skin	Skin - Not Sun Exposed (Suprapubic), Sun Exposed (Lower leg)	812
STAD	Stomach Adenocarcinoma	Stomach	36	414	Stomach	Stomach	174
TGCT	Testicular Germ Cell Tumors	testicular	0	154	Testis	Testis	165

THCA	Thyroid Carcinoma	Thyroid Gland	59	512	Thyroid	Thyroid	279
THYM	Thymoma		2	119	Blood	Blood	444
UCEC	Uterine Corpus Endometrioid Carcinoma	Endometrium	23	181	Uterus	Uterus	78
UCS	Uterine Carcinosarcoma	Uterus	0	57	Uterus	Uterus	78
UVM	Uveal Melanoma	Uveal	0	79	-	-	-

Supplementary Table 8. The basic information of enrolled patients in the glioma tissue microarray (ZL-BraG Sur1801).

Storage numbe	Pathology number	Tumor location	Histological type	Grade	Gender	Age	Surgical time	Survival time (months)	Vital status
N002	20156456	Right temporal lobe	Astrocytoma	II	Male	43	2015/6/3	20	Dead
N004	20160277	Left cerebellum	Pilocytic astrocytoma	II	Female	8	2016/3/1	9	Dead
N006	20162321	Left cerebellum	Oligodendrocytoma	II	Male	30	2016/11/1	19	Dead
N007	20162413	Double frontal corpus callosum	Astrocytoma	II	Female	49	2016/3/5	15	Dead
N010	20163816	Right temporal lobe	Oligodendrocytoma	II	Male	44	2016/4/21	22	Dead
N025	20172221	Not Otherwise Specified	Astrocytoma	II	Male	50	2017/11/8	4	Dead
N026	29172562	Cerebellum	Pilocytic astrocytoma	II	Male	11	2017/1/4	30	Alive
N027	21072694	Right temporal lobe	Oligodendrocytoma	II	Female	48	2017/2/26	29	Alive
N032	20173784	Right frontal lobe	Oligodendrocytoma	II	Female	30	2017/3/25	19	Dead
N049	20182507	Right corpus callosum	Astrocytoma	II	Female	49	2018/3/5	14	Dead
N058	20185377	Right frontal lobe	Astrocytoma	II	Female	36	2018/5/12	6	Dead

N060	20186040	Left temporal lobe	Diffuse astrocytoma	II	Male	58	2018/5/28	12	Dead
N061	20186264	Right frontotemporal	Oligodendrocytoma	II	Male	27	2018/6/14	13	Alive
N075	20153091	Left temporal lobe	Diffuse astrocytoma	II	Female	58	2015/2/25	21	Dead
N077	20153462	Right frontal lobe	Diffuse astrocytoma	II	Female	52	2015/3/4	16	Dead
N078	20153433	Left frontal lobe	Ganglioglioma	II	Male	50	2015/3/3	18	Dead
N080	20152669	Right frontal lobe	Astrocytoma	II	Female	45	2015/1/11	22	Dead
N081	20152514	Left temporal lobe	Oligodendrocytoma	II	Female	45	2015/1/24	38	Dead
N083	20151872	Left parietal lobe	Fibrous astrocytoma	II	Male	73	2015/1/19	29	Dead
N084	2152367	Right temporal lobe	Fibrous astrocytoma	II	Male	36	2015/1/25	23	Dead
N086	20151685	Right frontal lobe	Anaplastic astrocytoma	II	Male	42	2015/8/7	14	Dead
N087	13-0847	Right frontal lobe	Diffuse astrocytoma	II	Male	48	2013/9/7	22	Dead
N088	20145787	Left frontal lobe	Oligodendrocytoma	II	Female	52	2014/6/25	10	Dead
N090	20145027	Right parietal lobe	Oligodendrocytoma	II	Female	57	2014/3/24	11	Dead

N091	20144864	Left frontal lobe	Astrocytoma	II	Female	41	2014/3/10	15	Dead
N093	20143746	Left temporal lobe	Glioblastoma multiforme	II	Female	44	2014/7/27	4	Dead
N094	20143737	Right temporal lobe	Oligodendrocytoma	II	Male	29	2014/3/18	22	Dead
N097	21043138	Left frontal and temporal lobes	Glioblastoma multiforme	II	Male	46	2014/8/4	2	Dead
N099	20142126	Right frontal lobe	Astrocytoma	II	Male	43	2014/6/18	7	Dead
N100	20141961	Right frontal lobe	Anaplastic astrocytoma	II	Male	30	2014/7/9	15	Dead
N102	20141625	Left frontal-parietal lobe	Astrocytoma	II	Male	60	2014/7/6	12	Dead
N106	20140360	Left parietal lobe	Astrocytoma	II	Male	64	2014/4/2	11	Dead
N107	20140279	Right frontal lobe	Astrocytoma	II	Male	28	2014/1/14	8	Dead
N108	20140194	Left frontal lobe	Anaplastic astrocytoma	II	Female	50	2014/1/4	5	Dead
N119	20132967	Right temporal lobe	Oligodendrocytoma	II	Male	22	2013/8/4	1	Dead
N126	20134839	Left ventricle	Astrocytoma	II	Female	34	2013/4/22	3	Dead
N128	20135255	Right cerebellum	Anaplastic astrocytoma	II	Female	20	2013/5/10	7	Dead

N129	20132287	Right frontal lobe	Diffuse astrocytoma	II	Male	26	2013/1/17	3	Dead
N143	20110107	Right frontal lobe	Diffuse astrocytoma	II	Female	29	2011/4/25	7	Dead
N150	20111828	Cerebellum	Pilocytic astrocytoma	II	Male	17	2011/9/24	2	Dead
N153	20112167	cerebellum	Pilocytic astrocytoma	II	Male	15	2011/11/7	13	Dead
N155	20112316	Right temporal lobe	Glioblastoma multiforme	II	Male	61	2011/7/6	8	Dead
N161	20114143	Left temporal lobe	Oligodendrocytoma	II	Female	29	2011/3/24	24	Dead
N168	20122204	Right temporal lobe	Anaplastic oligodendroglioma	II	Male	46	2012/11/4	20	Dead
N169	20122417	Right temporal lobe	Diffuse astrocytoma	II	Male	32	2012/3/15	14	Dead
N174	12-3870	Right parietal lobe	Oligodendrocytoma	II	Male	39	2012/7/25	0	Dead
N176	20125147	Right ventricle	Anaplastic astrocytoma	II	Male	21	2012/4/28	8	Dead
N182	13-1248	Right temporal lobe	Diffuse astrocytoma	II	Male	72	2013/11/7	2	Dead
N188	13-4765	Frontal lobe	Diffuse astrocytoma	II	Male	42	2013/7/2	4	Dead
N191	16-4477	Left parietal lobe	Diffuse astrocytoma	II	Male	68	2016/4/14	5	Dead

N194	16-641	Right frontal lobe	Anaplastic astrocytoma	II	Female	55	2016/5/28	13	Dead
N195	16-748	Right frontal lobe	Anaplastic astrocytoma	II	Male	43	2016/6/23	21	Dead
N196	16-1743	Right temporal lobe	Diffuse astrocytoma	II	Male	37	2016/8/5	14	Dead
N201	15-2764	Right frontal lobe	Oligodendrocytoma	II	Female	45	2016/4/28	9	Dead
N202	15-3619	Left temporal lobe	Oligodendrocytoma	II	Female	47	2015/3/2	8	Dead
N021	20171213	Right temporal lobe	Anaplastic astrocytoma	III	Female	64	2017/8/24	8	Dead
N023	20172028	Left frontal lobe	Anaplastic astrocytoma	III	Female	53	2017/4/8	14	Dead
N044	20181412	Left frontal lobe	Anaplastic oligodendroglioma	III	Female	40	2018/11/5	8	Alive
N059	20185632	Corpus callosum	Glioblastoma multiforme	III	Male	57	2018/5/7	8	Dead
N063	20190266	Left temporal lobe	Glioblastoma multiforme	III	Male	54	2019/2/11	5	Alive
N067	20190831	Left frontal lobe	Glioblastoma multiforme	III	Male	51	2019/6/25	1	Alive
N073	20165929	Double frontal corpus callosum	Glioblastoma multiforme	III	Male	22	2016/5/25	17	Dead
N074	20153880	Left frontal lobe	Anaplastic astrocytoma	III	Male	27	2015/3/10	30	Dead

N076	20133087	Left frontal lobe	Anaplastic astrocytoma	III	Female	20	2013/7/8	7	Dead
N082	20151890	Right parietal lobe	Fibrous astrocytoma	III	Female	41	2015/1/14	0	Dead
N087	20150864	Left temporal lobe	Gliosarcoma	III	Female	58	2015/7/24	23	Dead
N089	20145029	Left temporal lobe	Anaplastic astrocytoma	III	Male	16	2014/4/26	9	Dead
N092	20143972	Right parietal lobe	Anaplastic astrocytoma	III	Female	40	2014/2/26	5	Dead
N101	20141949	Right parietal lobe	Glioblastoma multiforme	III	Female	62	2014/9/14	16	Dead
N118	20132946	Right frontal lobe	Anaplastic astrocytoma	III	Female	41	2013/9/12	13	Dead
N121	20153087	Right frontal lobe	Anaplastic astrocytoma	III	Female	70	2015/2/23	26	Dead
N123	20133316	Left temporal lobe	Anaplastic astrocytoma	III	Male	50	2013/7/1	17	Dead
N125	20133546	Right frontal lobe	Glioblastoma multiforme	III	Female	53	2013/7/8	16	Dead
N139	10-4322	Left frontal lobe	Anaplastic oligodendroglioma	III	Male	54	2010/3/28	39	Dead
N140	10-5014	Corpus callosum	Glioblastoma multiforme	III	Male	58	2010/4/8	7	Dead
N152	20112036	Left temporal lobe	Anaplastic astrocytoma	III	Male	39	2011/5/24	13	Dead

N157	20112932	frontal and parietal lobes	Diffuse astrocytoma	III	Male	45	2011/9/14	13	Dead
N193	16-4360	Right parietal lobe	Glioblastoma multiforme	III	Male	57	2016/3/28	29	Dead
N197	16-4529	Right frontal lobe	Anaplastic astrocytoma	III	Female	68	2016/4/1	16	Dead
N200	16-5316	Left frontal lobe	Anaplastic astrocytoma	III	Male	27	2016/4/21	8	Dead
N203	16-1998	Right frontal lobe	Diffuse astrocytoma	III	Female	45	2016/9/25	27	Dead
N024	20172201	Right frontal lobe	Anaplastic astrocytoma	II-III	Male	35	2017/11/5	3	Dead
N130	20132572	Right triangle	Glioblastoma multiforme	II-III	Female	59	2013/4/1	7	Dead
N170	20123306	Right frontal lobe	Anaplastic astrocytoma	II-III	Male	41	2012/2/21	25	Dead
N001	20156219	Right temporal lobe	Glioblastoma multiforme	IV	Male	55	2015/6/5	41	Dead
N003	20157681	Left frontal-parietal lobe	Glioblastoma multiforme	IV	Male	35	2015/6/9	21	Dead
N005	20160729	Right occipital lobe	Glioblastoma multiforme	IV	Male	51	2016/7/1	17	Dead
N008	20163223	Double frontal corpus callosum	Glioblastoma multiforme	IV	Male	60	2016/3/18	13	Dead
N009	20163768	Right temporal lobe	Glioblastoma multiforme	IV	Male	67	2016/3/20	18	Dead

N011	20164990	Left frontal lobe	Glioblastoma multiforme	IV	Female	22	2016/4/7	10	Dead
N012	20165735	Right parietal lobe	Glioblastoma multiforme	IV	Male	57	2016/5/20	12	Dead
N013	20165739	Frontal lobe	Glioblastoma multiforme	IV	Male	59	2016/5/15	23	Dead
N014	20166006	Left parietal lobe	Glioblastoma multiforme	IV	Female	73	2016/6/8	32	Dead
N015	20166573	Left temporal lobe	Glioblastoma multiforme	IV	Male	52	2016/6/15	27	Dead
N017	20170257	Right frontal lobe	Glioblastoma multiforme	IV	Male	64	2017/2/1	12	Dead
N018	21070304	Right occipital lobe	Glioblastoma multiforme	IV	Female	16	2017/3/6	6	Dead
N019	21070533	Right temporal lobe	Glioblastoma multiforme	IV	Male	64	2017/5/8	10	Dead
N020	20171006	Left temporal parietal lobe	Glioblastoma multiforme	IV	Female	38	2017/9/12	8	Dead
N022	20171794	ventricle of the brain	Glioblastoma multiforme	IV	Male	69	2017/4/17	0	Dead
N028	20173041	Left frontal lobe	Glioblastoma multiforme	IV	Male	56	2017/3/1	10	Dead
N029	20173175	Right frontal lobe	Glioblastoma multiforme	IV	Female	38	2017/3/5	14	Dead
N030	20173348	Left parietal lobe	Glioblastoma multiforme	IV	Male	50	2017/3/8	17	Dead

N031	20173471	Right temporal lobe	Glioblastoma multiforme	IV	Male	57	2017/3/15	28	Alive
N033	20174062	Left frontal lobe	Glioblastoma multiforme	IV	Male	82	2017/4/4	21	Dead
N034	20174441	Right frontal lobe	Glioblastoma multiforme	IV	Male	31	2017/4/2	10	Dead
N035	20175160	Right parietal lobe	Glioblastoma multiforme	IV	Female	42	2017/4/23	18	Dead
N037	21075707	Right temporal lobe	Glioblastoma multiforme	IV	Male	54	2017/5/19	14	Dead
N038	20175861	Right temporal lobe	Glioblastoma multiforme	IV	Male	54	2017/5/1	16	Dead
N039	20180109	Right ventricle	Glioblastoma multiforme	IV	Male	57	2018/1/4	6	Dead
N040	20180374	Left parietal lobe	Glioblastoma multiforme	IV	Male	72	2018/3/14	16	Alive
N041	20180655	Left frontal lobe	Glioblastoma multiforme	IV	Female	41	2018/6/11	9	Dead
N042	20180773	Right frontal lobe	Glioblastoma multiforme	IV	Female	34	2018/7/4	13	Dead
N043	20180823	Left frontal lobe	Glioblastoma multiforme	IV	Male	74	2018/8/14	3	Dead
N046	20181489	Left temporal lobe	Glioblastoma multiforme	IV	Male	66	2018/11/8	2	Dead
N047	20181626	Right temporal lobe	Glioblastoma multiforme	IV	Male	72	2018/7/4	9	Dead

N048	20182027	Right parietal lobe	Glioblastoma multiforme	IV	Male	54	2018/2/24	9	Dead
N050	20182553	Right frontal lobe	Glioblastoma multiforme	IV	Female	68	2018/3/7	16	Alive
N051	20182768	Right temporal lobe	Glioblastoma multiforme	IV	Male	35	2018/3/14	13	Dead
N052	20182770	Right temporal lobe	Glioblastoma multiforme	IV	Male	66	2018/2/24	17	Dead
N053	20183140	Left temporal lobe	Glioblastoma multiforme	IV	Female	65	2018/3/10	5	Dead
N054	20123423	Left temporal lobe	Glioblastoma multiforme	IV	Male	54	2012/3/1	8	Dead
N056	20184929	Right frontal lobe	Glioblastoma multiforme	IV	Male	45	2018/4/18	14	Dead
N062	20190128	Right temporal lobe	Glioblastoma multiforme	IV	Male	74	2019/1/4	0	Dead
N064	20190415	Left triangle	Glioblastoma multiforme	IV	Female	64	2019/4/2	3	Alive
N065	20190471	Right occipital lobe	Glioblastoma multiforme	IV	Male	76	2019/4/5	3	Dead
N066	20190807	Right temporal lobe	Glioblastoma multiforme	IV	Female	63	2019/6/20	1	Alive
N068	20190893	Right occipital lobe	Glioblastoma multiforme	IV	Male	56	2019/7/1	1	Alive
N079	20153432	Right temporal lobe	Glioblastoma multiforme	IV	Female	53	2015/4/7	20	Dead

N085	20156977	Right temporal lobe	Glioblastoma multiforme	IV	Female	64	2015/6/15	7	Dead
N095	20155203	Not Otherwise Specified	Glioblastoma multiforme	IV	Female	44	2015/3/3	17	Dead
N096	20150876	Left frontal lobe	Glioblastoma multiforme	IV	Male		2015/7/18	4	Dead
N098	20142384	Left frontal lobe	Glioblastoma multiforme	IV	Male	56	2014/7/12	11	Dead
N103	20140686	Left inferior parietal lobe	Glioblastoma multiforme	IV	Male	52	2014/5/27	1	Dead
N104	20140703	Left temporal lobe	Glioblastoma multiforme	IV	Male	51	2014/6/25	5	Dead
N105	20140529	Left temporal lobe	Glioblastoma multiforme	IV	Male	70	2014/5/22	9	Dead
N109	20130178	Left temporal lobe	Glioblastoma multiforme	IV	Female	46	2013/10/16	4	Dead
N110	20130179	Right temporal and parietal	Glioblastoma multiforme	IV	Female	78	2013/8/4	3	Dead
N111	20130563	Right temporal lobe	Glioblastoma multiforme	IV	Male	64	2013/7/12	9	Dead
N112	20130776	Temporal lobe	Glioblastoma multiforme	IV	Male	50	2013/5/23	2	Dead
N113	20131020	frontal and parietal lobes	Glioblastoma multiforme	IV	Male	46	2013/9/14	2	Dead
N114	20131179	Left parietal lobe	Glioblastoma multiforme	IV	Male	55	2013/10/26	17	Dead

N115	20131305	Left temporal lobe	Glioblastoma multiforme	IV	Male	68	2013/8/4	6	Dead
N116	20131647	Left parietal lobe	Glioblastoma multiforme	IV	Male	77	2013/12/4	15	Dead
N120	20133064	frontal and parietal lobes	Glioblastoma multiforme	IV	Female	50	2013/5/8	6	Dead
N122	20133115	Left frontal lobe	Glioblastoma multiforme	IV	Male	67	2013/6/2	11	Dead
N127	20135242	Left frontal and temporal lobes	Glioblastoma multiforme	IV	Male	61	2013/4/21	4	Dead
N133	10-678	Left frontal lobe	Glioblastoma multiforme	IV	Male	58	2010/5/28	10	Dead
N134	10-1327	Right temporal lobe	Glioblastoma multiforme	IV	Male	67	2010/6/4	1	Dead
N135	10-2419	Right occipital lobe	Glioblastoma multiforme	IV	Male	56	2010/5/27	6	Dead
N136	10-2459	Left temporal lobe	Glioblastoma multiforme	IV	Female	75	2010/2/28	6	Dead
N138	10-3871	Left frontal lobe	Glioblastoma multiforme	IV	Female	43	2010/3/14	22	Dead
N141	10-5471	Right temporal lobe	Glioblastoma multiforme	IV	Male	36	2010/4/28	10	Dead
N142	10-5741	Right occipital lobe	Glioblastoma multiforme	IV	Male	78	2010/4/16	0	Dead
N146	20111317	Left temporal lobe	Oligodendrocytoma	IV	Male	77	2011/9/1	16	Dead

N148	20111531	Right parietal lobe	Glioblastoma multiforme	IV	Male	73	2011/8/5	0	Dead
N149	20111814	Right frontal lobe	Glioblastoma multiforme	IV	Female	59	2011/8/1	7	Dead
N151	20111870	Right frontal lobe	Glioblastoma multiforme	IV	Male	58	2011/7/21	4	Dead
N154	20112315	Right parietal lobe	Glioblastoma multiforme	IV	Female	63	2011/6/10	5	Dead
N158	20112994	Left temporal lobe	Glioblastoma multiforme	IV	Male	67	2011/4/5	3	Dead
N159	20113927	Right thalamus	Glioblastoma multiforme	IV	Female	33	2011/1/14	0	Dead
N163	20120443	Left frontal lobe	Glioblastoma multiforme	IV	Male	43	2012/3/15	16	Dead
N164	12-0713	Right frontal lobe	Glioblastoma multiforme	IV	Female	34	2012/6/25	8	Dead
N166	20121610	Right temporal lobe	Glioblastoma multiforme	IV	Male	49	2012/9/14	2	Dead
N167	12-2041	Right parietal lobe	Glioblastoma multiforme	IV	Male	55	2012/1/8	3	Dead
N171	20183423	Left temporal lobe	Glioblastoma multiforme	IV	Female	74	2018/3/22	12	Dead
N173	20123869	Right frontal lobe	Glioblastoma multiforme	IV	Male	61	2012/4/18	16	Dead
N175	12-4961	Right frontal lobe	Glioblastoma multiforme	IV	Male	48	2012/4/18	9	Dead

N178	12-5768	Right temporal lobe	Glioblastoma multiforme	IV	Male	67	2012/4/11	13	Dead
N179	13-0671	Left triangle	Glioblastoma multiforme	IV	Male	53	2013/5/14	15	Dead
N181	13-1158	Left temporal parietal lobe	Glioblastoma multiforme	IV	Male	50	2013/10/24	30	Dead
N183	13-1257	Left ventricle	Glioblastoma multiforme	IV	Male	65	2013/11/8	1	Dead
N184	13-1317	Left triangle	Glioblastoma multiforme	IV	Female	66	2013/8/4	12	Dead
N185	13-1349	Right corpus callosum	Glioblastoma multiforme	IV	Male	68	2013/10/25	4	Dead
N186	13-1589	Left parietal lobe	Glioblastoma multiforme	IV	Male	58	2013/8/6	2	Dead
N187	13-2579	Left parietal lobe	Glioblastoma multiforme	IV	Female	68	2013/8/7	12	Dead
N189	12-4951	Right frontal lobe	Glioblastoma multiforme	IV	Male	71	2012/3/25	28	Dead
N190	13-6141	Left temporal lobe	Glioblastoma multiforme	IV	Male	62	2013/5/21	21	Dead
N192	16-7528	Right occipital lobe	Glioblastoma multiforme	IV	Female	55	2016/6/8	2	Dead
N198	16-7805	Left frontal lobe	Glioblastoma multiforme	IV	Male	59	2016/6/5	26	Dead
N199	15-7208	Right occipital lobe	Glioblastoma multiforme	IV	Female	45	2015/6/5	19	Dead

Supplementary Table 9. The basic information of enrolled patients in the glioma tissue microarray (HBra-Gli060PG-01).

Sample	Tumor location	Histological type	Grade	Gender	Age	Survival time (months)	Vital status	Primary organ or not	Metastasis or not
N0005P0101	white matter	Normal	——	Male	Adult	Not Available	Not Available	——	——
N0007P0104	white matter	Normal	——	Male	Adult	Not Available	Not Available	——	——
N0008P0100	white matter	Normal	——	Male	Adult	Not Available	Not Available	——	——
P01A0097	Right frontal lobe	Astrocytoma	II	Male	55	Not Available	Not Available	Yes	No
P01A0108	Left lateral ventricle	Astrocytoma	II	Female	43	Not Available	Not Available	Yes	No
P01A0110	Right frontal lobe	Astrocytoma	II	——	——	Not Available	Not Available	Yes	No
P01A0052	Right frontal lobe	Astrocytoma	II	Female	34	Not Available	Not Available	Yes	No
P01A0055	Left temporal region	Astrocytoma	II	Female	30	Not Available	Not Available	Yes	No
P01A0056	Right temporal lobe	Astrocytoma	II	Male	39	Not Available	Not Available	Yes	No
P01A0070	Left frontal lobe	Astrocytoma	II	Female	58	Not Available	Not Available	Yes	No
P01A0114	Right basal ganglia	Astrocytoma	II	Female	52	Not Available	Not Available	Yes	No

P01A0066	Left frontal lobe	Diffuse astrocytoma	II	Female	44	Not Available	Not Available	Yes	No
P01A0094	Right frontal lobe	Diffuse astrocytoma	II	Male	27	Not Available	Not Available	Yes	No
P01A0200	Right frontal lobe	Diffuse astrocytoma	II	Male	44	Not Available	Not Available	Yes	No
P01A0206	Right frontal lobe	Diffuse astrocytoma	II	Female	43	Not Available	Not Available	Yes	No
P01A0106	Right frontal lobe	Astrocytoma	III	Male	61	Not Available	Not Available	Yes	No
P01A0079	Right parietal lobe	Anaplastic astrocytoma	III	Female	30	Not Available	Not Available	Yes	No
P01A0080	Left frontal lobe	Anaplastic astrocytoma	III	Male	51	Not Available	Not Available	Yes	No
P01A0092	Right parietal lobe	Anaplastic astrocytoma	III	Female	36	Not Available	Not Available	Yes	No
P01A0109	Left frontal lobe	Anaplastic astrocytoma	III	Female	57	Not Available	Not Available	Yes	No
P01A0084	Left frontal lobe	Anaplastic astrocytoma	III	Female	73	Not Available	Not Available	Yes	No
P01A0085	Right temporal lobe	Anaplastic astrocytoma	III	Female	50	Not Available	Not Available	Yes	No
P01A0126	Left frontal lobe	Anaplastic astrocytoma	III	——	——	Not Available	Not Available	Yes	No

P01A0196	Left frontal lobe	Anaplastic astrocytoma	III	Male	31	Not Available	Not Available	Yes	No
P01A0124	Left superior parietal lobe	Anaplastic astrocytoma	III	——	——	Not Available	Not Available	Yes	No
P01A0197	Right frontal lobe	Anaplastic astrocytoma	III	Male	56	Not Available	Not Available	Yes	No

Supplementary Table 10. Detailed chromosomal distribution of the methylation probes associated with PLEKHA4.

Probe	Chromosome	Start	End	Distance from TSS	CpG position	Strand
cg04777726	chr19	48837232	48837281	31336	Island	-
cg16594139	chr19	48837268	48837317	31300	Island	+
cg26267310	chr19	48837287	48837336	31281	Island	+
cg19024700	chr19	48837509	48837558	31059	Island	-
cg15549821	chr19	48838845	48838894	29723	S_Shore	-
cg18689573	chr19	48841161	48841210	27407	S_Shelf	+
cg25261547	chr19	48860063	48860112	8495		+
cg06705122	chr19	48867773	48867822	795		+
cg06339706	chr19	48868029	48868078	539		-
cg01093065	chr19	48868795	48868844	-227	N_Shelf	+
cg23002721	chr19	48869352	48869401	-781	N_Shelf	-

Supplementary Table 11. Correlation analysis between PLEKHA4 and various gene markers of immune cells in CGGA-LGG (mRNAseq_325) cohort.

Description	Gene markers	Cor	*P*
CD8+ T cell	CD8A	0.39	***
	CD8B	0.44	***
T cell (general)	CD3D	0.57	***
	CD3E	0.63	***
	CD2	0.59	***
	CD3G	0.54	***
	CD4	0.64	***
B cell	CD19	0.38	***
	CD79A	0.32	***
	CD79B	0.37	***
	MS4A1	0.28	***
Monocyte	CD86	0.57	***
	CD115 (CSF1R)	0.40	***
TAM	CCL2	0.47	***
	CD68	0.66	***
	IL10	0.46	***
M1 Macrophage	TNOS (NOS2)	-0.11	**
	PTGS2	0.18	**
M2 Macrophage	CD163	0.59	***
	VSIG4	0.47	***
	TGFB1	0.65	***
Neutrophils	CD11b (ITGAM)	0.47	***
	CCR7	0.32	***
	FCGR3B	0.26	***
	CXCR2	0.23	**
NK cell	KLRF1	0.12	0.10
	GNLY	0.40	***
	NKG7	0.56	***
	KLRD1	0.28	***
Dendritic cell	HLA-DPB1	0.66	***
	HLA-DQB1	0.35	***
	HLA-DRA	0.71	***
	HLA-DPA1	0.68	***
	CD11C (ITGAX)	0.46	***
Th1	T-bet (TBX21)	0.40	***
	STAT1	0.61	***
Th2	GATA3	0.37	***
	STAT6	0.43	***
	STAT5A	0.61	***
	IL6	0.53	***
Tfh	BCL6	0.29	***
	CXCR5	0.10	0.16

Th17	STAT3	0.62	***
	IL17A	0.36	***
Treg	FOXP3	0.06	0.45
	STAT5B	0.15	*
Tex	PD-1 (PDCD1)	0.54	***
	CTLA4	0.29	***
	LAG3	0.43	***
	TIM-3 (HAVCR2)	0.58	***
Mast cells	TPSB2	0.26	***
	TPSAB1	0.25	***
	HDC	0.26	***

TAM tumor-associated macrophage, *Th* T helper cell, *Tfh* Follicular helper T cell, *Treg* regulatory T cell, *Tex* exhausted T cell, *Cor* R value of Spearman's correlation. *< 0.05, **< 0.01, ***< 0.001.
